# Supplementary material for: A negative-solvatochromic fluorescent probe for visualizing intracellular distributions of fatty acid metabolites
Source: Nat Commun. 2022 May 9;13:2533. doi: 10.1038/s41467-022-30153-6 (PMC9085894; doi:10.1038/s41467-022-30153-6)
Supplement: Supplementary file 1 — Supplementary Information [file 41467_2022_30153_MOESM1_ESM.pdf]

Supplementary Information

A negative-solvatochromic fluorescent fatty acid probe for visualizing intracellular distributions of fatty acid metabolites

Keiji Kajiwara[1], Hiroshi Osaki[1], Steffen Greßies[2], Keiko Kuwata[3], Ju Hyun Kim[2], Tobias Gensch[2], Yoshikatsu Sato[3], Frank Glorius*[,2], Shigehiro Yamaguchi*[,1,3], Masayasu Taki*[,3]

[1] *Department of Chemistry, Graduate School of Science, and Integrated Research Consortium on Chemical Sciences (IRCCS), Nagoya University, Furo, Chikusa, Nagoya 464-8602, Japan*

[2] *Organisch-Chemisches Institut, Westfälische Wilhelms-Universität Münster, Corrensstraße 40, 48149 Münster, Germany*

[3] *Institute of Transformative Bio-Molecules (WPI-ITbM), Nagoya University, Furo, Chikusa, Nagoya 464-8601, Japan*

Supplementary Fig. 1	UV-Vis absorption and emission spectra of dyes **2-6**
Supplementary Fig. 2	Theoretical calculations of the AP skeleton
Supplementary Fig. 3	Fluorescence spectra of AP-Me in pH-buffered solutions
Supplementary Fig. 4	Cell viability results of HepG2 cells treated with AP-C12
Supplementary Figs. 5-7	LC/MS chart of AP-C12 metabolites
Supplementary Fig. 8	Confocal images of HepG2 cells incubated with AP-C6
Supplementary Figs. 9-10	LC/MS chart of BODIPY 558/568-C12 metabolites
Supplementary Fig. 11	A schematic diagram of fatty acid metabolic pathways
Supplementary Fig. 12	Size and number of LDs treated with/without etomoxir
Supplementary Fig. 13	Number of nuclear LDs treated with/without etomoxir
Supplementary Figs. 14-25	Confocal images
Supplementary Fig. 14	Control (HBSS-starved HepG2 cells)
Supplementary Fig. 15	Treatment with bafilomycin A1
Supplementary Fig. 16	Cells expressing GFP-LC3 after treatment with inhibitors
Supplementary Fig. 17	Treatment with 3-methyladenine
Supplementary Fig. 18	Treatment with diethylumbelliferyl phosphate
Supplementary Fig. 19	Pulse-chase assay with AP-C12
Supplementary Fig. 20	Treatment with rapamycin
Supplementary Fig. 21	Atg5$^{-/-}$ MEFs incubated under HBSS-starved conditions
Supplementary Fig. 22	Atg5$^{-/-}$ MEFs (bafilomycin A1)
Supplementary Fig. 23	Atg5$^{+/+}$ MEFs (HBSS)
Supplementary Fig. 24	Atg5$^{+/+}$ MEFs (bafilomycin A1)
Supplementary Fig. 25	MEFs expressing GFP-LC3
Supplementary Fig. 26	Synthetic scheme for AP-C6 and AP-C12
Supplementary Figs. 27-42	NMR spectra
Supplementary Table 1	Photophysical Data for AP-Me and dye **2**–**6**.
Supplementary Note 1	Synthesis

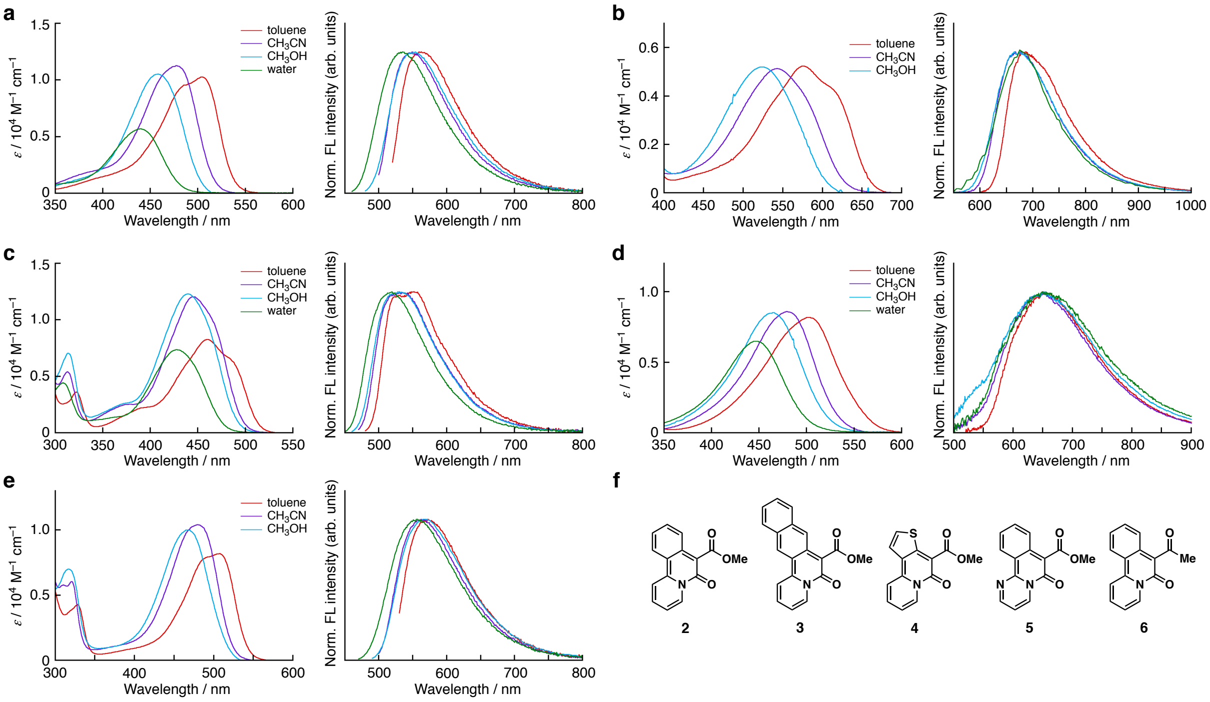

Supplementary Fig. 1. UV-Vis absorption (left) and emission (right) spectra of dye **2** (a), **3** (b), **4** (c), **5** (d), **6** (e) measured in an indicated solvent. Source data for this figure are provided as a Source Data file.

a

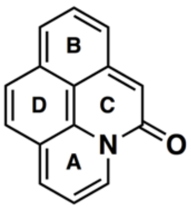

	toluene	CH_2Cl_2	H_2O
A	-10.86	-10.91	-10.94
B	-10.90	-11.13	-11.25
C	-4.69	-4.62	-4.58
D	-4.72	-4.83	-4.91

b

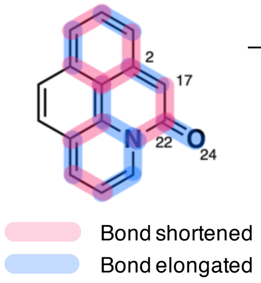

	toluene	CH_2Cl_2	H_2O
C22-O24	1.2418	1.2482	1.2511
C22-C17	1.4003	1.3970	1.3957
C17-C2	1.4097	1.4126	1.4139

Supplementary Fig. 2. Theoretical calculations of the AP skeleton. **a**, Calculated NICS (0) values in various solvents at the HF/6-31+G* level. **b**, Bond length in various solvents (B3LYP/6-31 + G*; PCM).

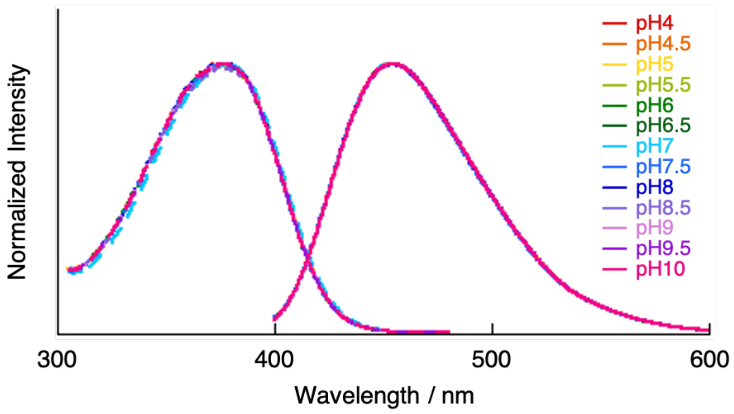

Supplementary Fig. 3. Excitation (dashed) and emission (solid) spectra of AP-Me in aqueous buffer solutions at various pH values. Source data for this figure are provided as a Source Data file.

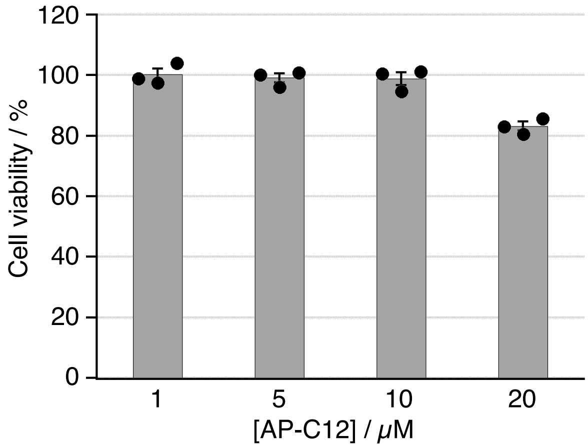

Supplementary Fig. 4. Cell viability results of HepG2 cells treated with AP-C12 for 24 h by MTT assay. The results are expressed as percentages of the dye-free controls. The results shown are the mean ± SEM of three separate experiments done in duplicate. Source data for this figure are provided as a Source Data file.

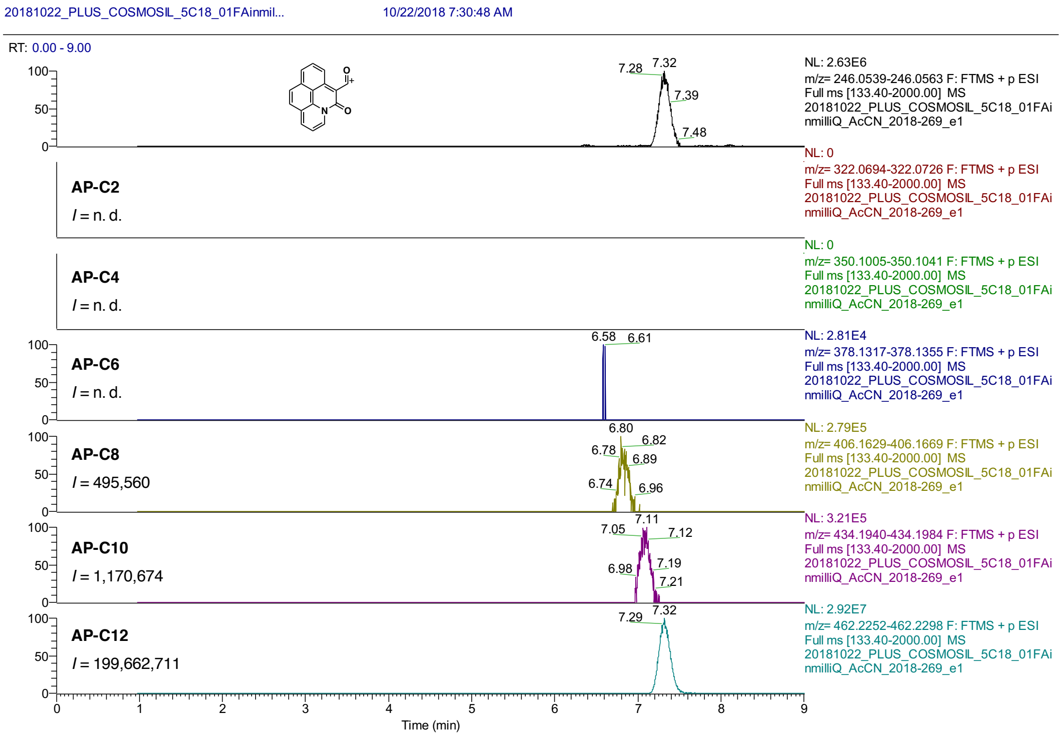

Supplementary Fig. 5. LC/MS chart of organic layer extracted from HepG2 cells treated with 5 μM AP-C12 for 1 h. *I* value means the integrated peak intensity.

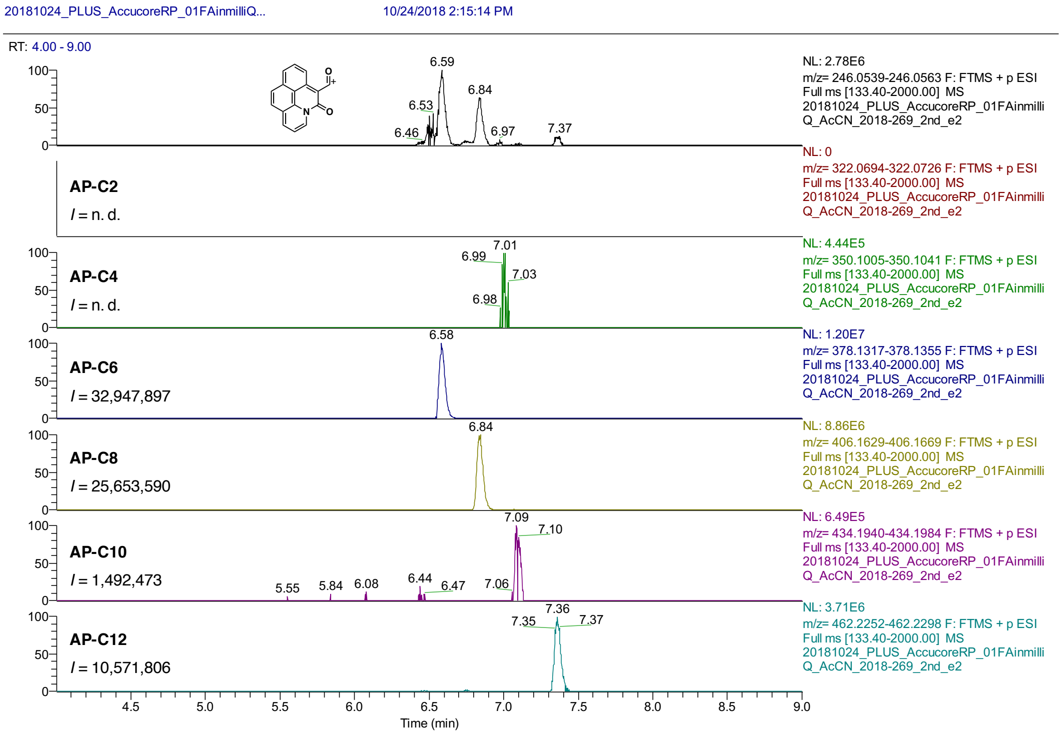

Supplementary Fig. 6. LC/MS chart of aqueous layer extracted from HepG2 cells treated with 5 μM AP-C12 for 1 h. *I* value means the integrated peak intensity.

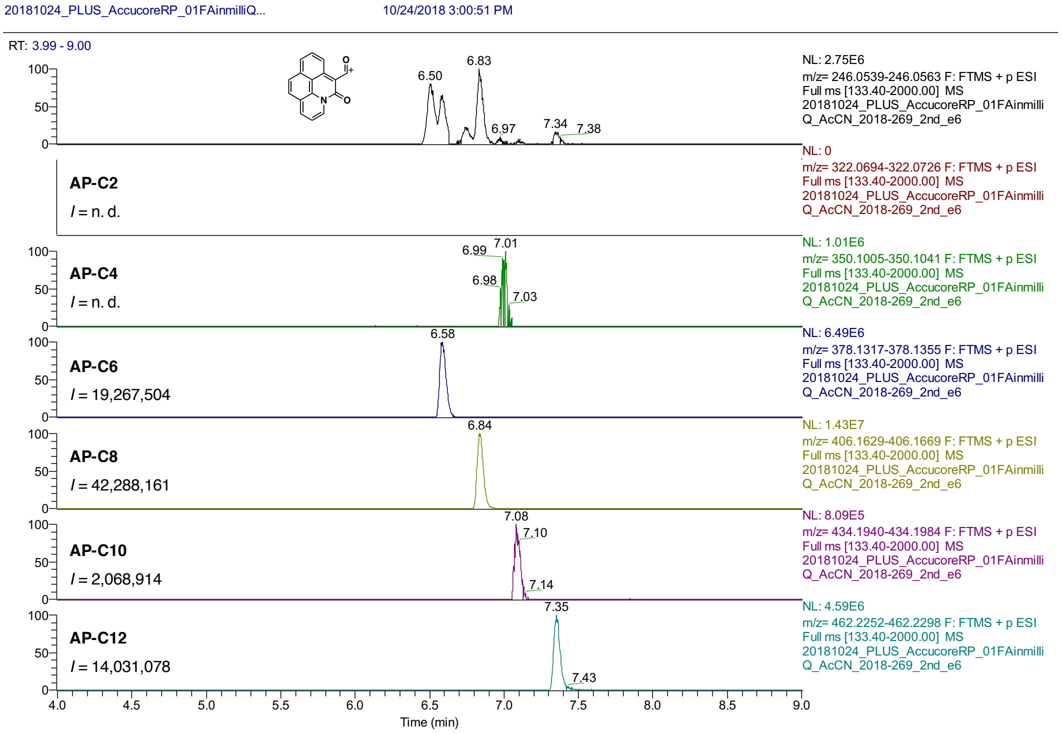

Supplementary Fig. 7. LC/MS chart of aqueous layer extracted from the medium where HepG2 cells were incubated with 5 μM AP-C12 for 1 h. *I* value means the integrated peak intensity.

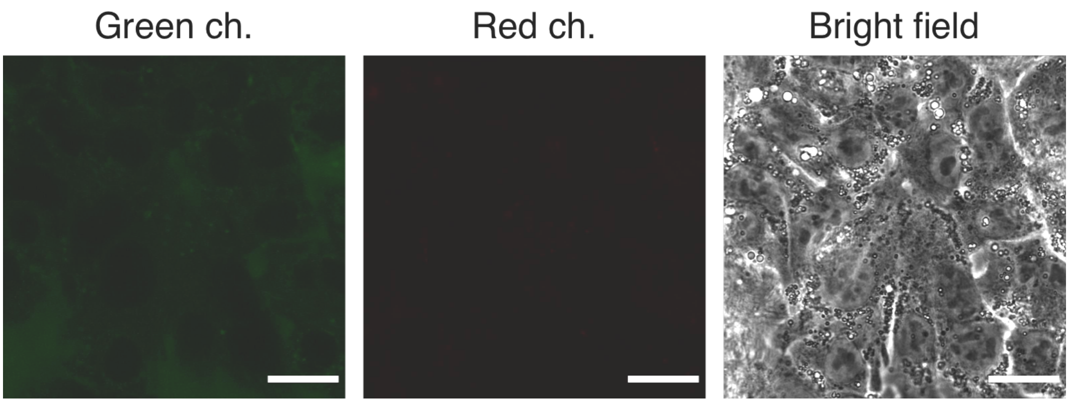

Supplementary Fig. 8. Confocal images of HepG2 cells incubated with 5 μM AP-C6 for 1 h, followed by detecting in the green (left) and red (center) channels. The bright field image is shown in right. Scale bar is 20 μm.

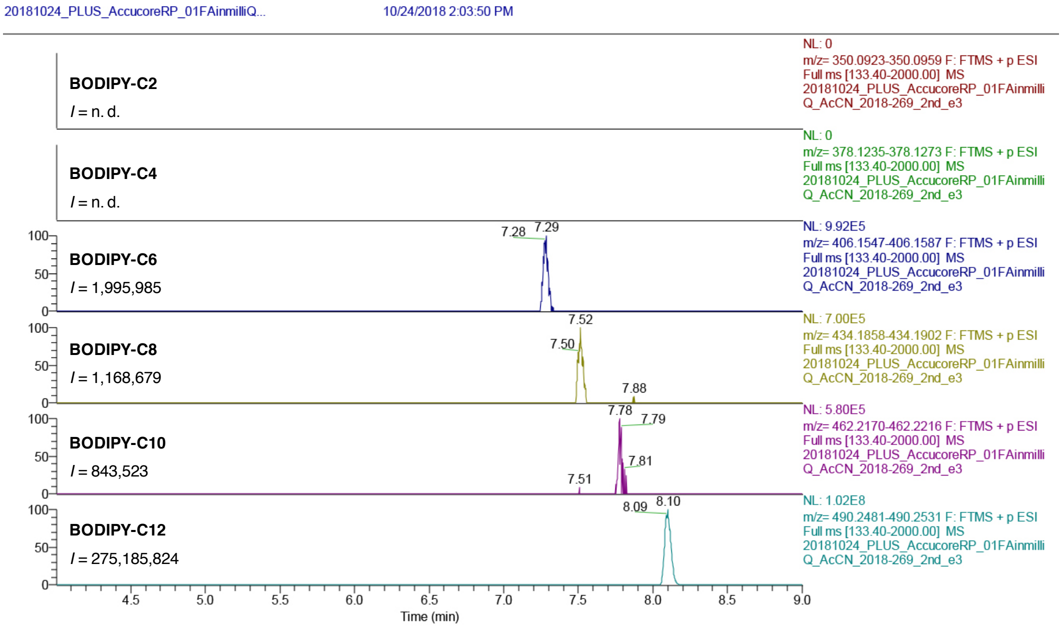

Supplementary Fig. 9. LC/MS chart of organic layer extracted from HepG2 cells treated with 5 μM BODIPY 558/568-C12 for 1 h. *I* value means the integrated peak intensity.

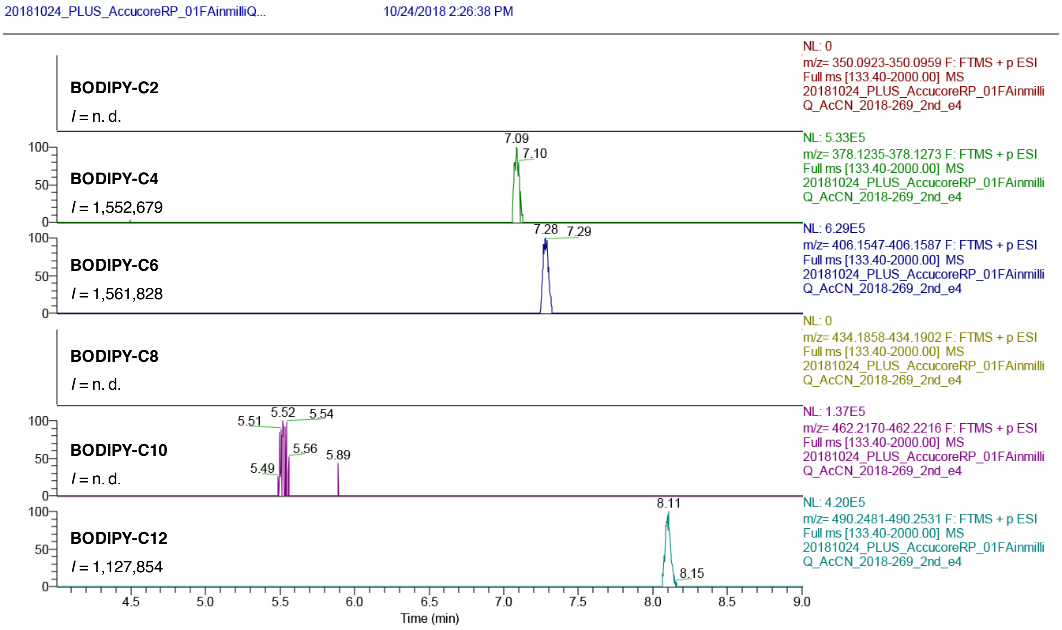

Supplementary Fig. 10. LC/MS chart of aqueous layer extracted from HepG2 cells treated with 5 μM BODIPY 558/568-C12 for 1 h. *I* value means the integrated peak intensity.

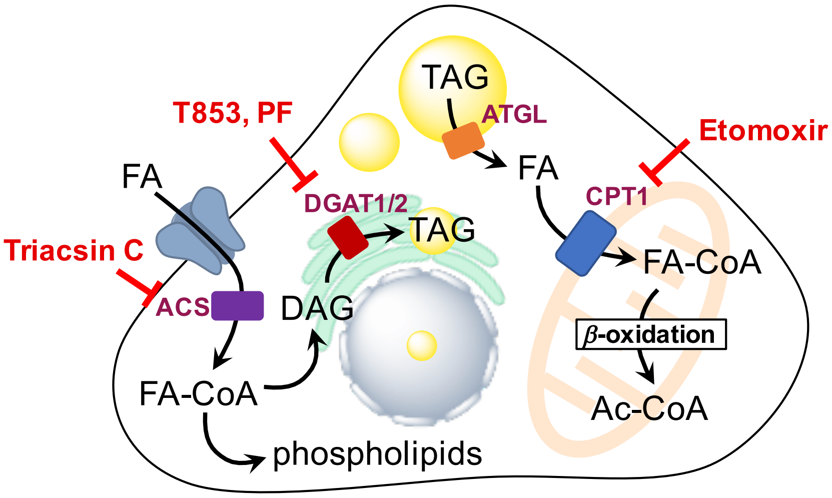

Supplementary Fig. 11. A schematic diagram of fatty acid metabolic pathways in a cell and known inhibitors for the fatty acid metabolism.

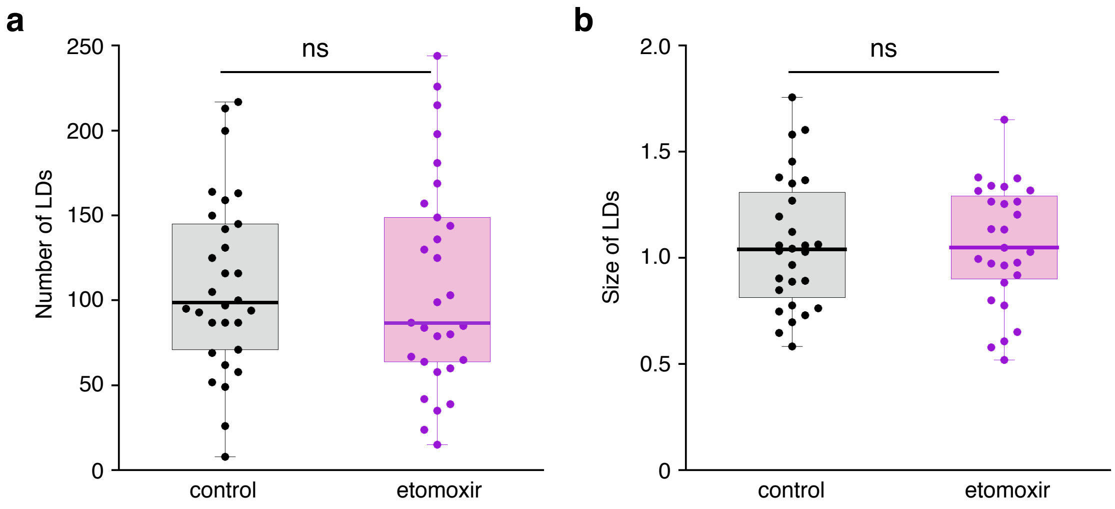

Supplementary Fig. 12. a) The number of LDs in HepG2 cells treated with/without etomoxir per cell (n = 30 and 29 cells for control and etomoxir treatment, respectively). P = 0.979. b) Average size of LDs (μm^2) in HepG2 cells treated with/without etomoxir per cell (n = 28 and 27 cells for control and etomoxir treatment, respectively). P = 0. 982. Box and whisker plots show the median (center line), first and third quartiles (box edges). The P values were calculated by unpaired two-tailed Student's t-test. NS, not significant. Source data for this figure are provided as a Source Data file.

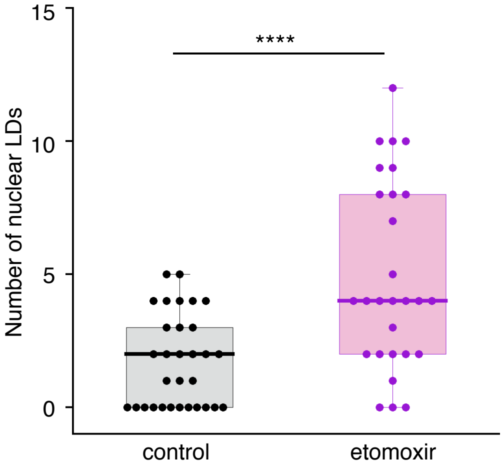

Supplementary Fig. 13. The number of nuclear LDs in HepG2 cells treated with/without etomoxir per cell. $n = 31$ cells for control and $n = 28$ cells for etomoxir-treated. ****$P = 0.00010$ was calculated by unpaired two-tailed Student's t-test. Source data for this figure are provided as a Source Data file.

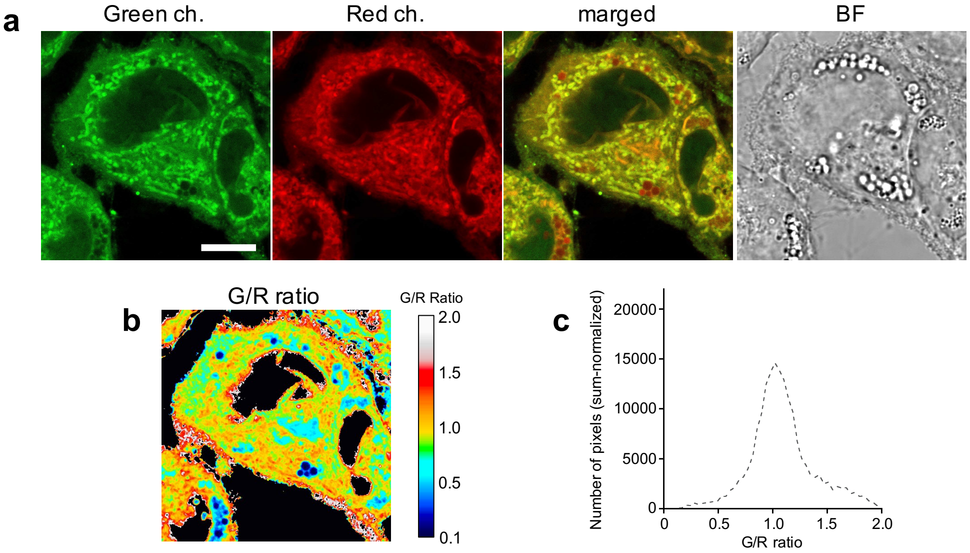

Supplementary Fig. 14. a, Confocal images of HepG2 cells incubated in HBSS+ containing 5 μM AP-C12 for 6 h. Images were recorded in the green and red channels, which were then superimposed. BF means its bright-filed image. Scale bar is 10 μm. **b,** The fluorescence ratio was calculated by dividing the intensities of the both channels (G/R). **c,** Distribution histogram of the G/R ratios. Source data for this figure are provided as a Source Data file.

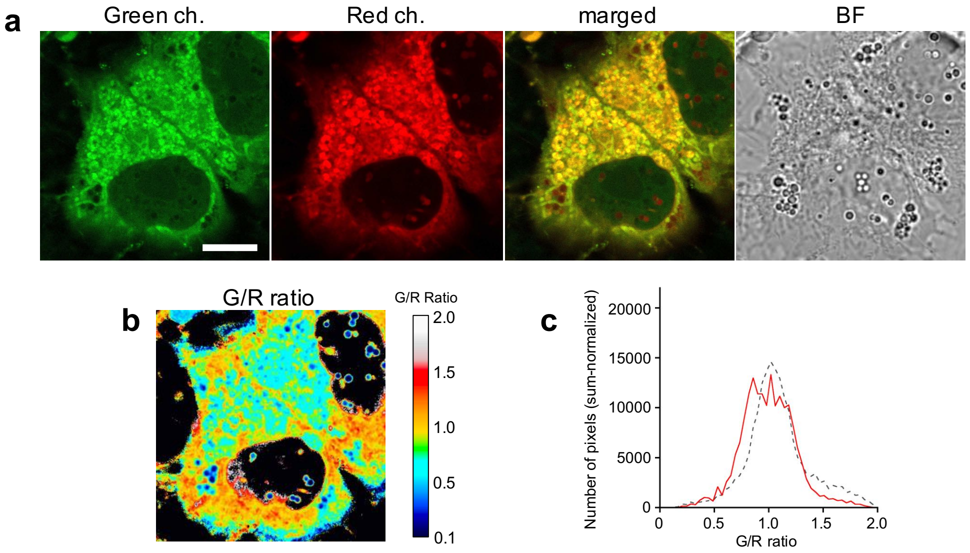

Supplementary Fig. 15. a, Confocal images of HepG2 cells incubated in HBSS+ containing 5 μM AP-C12 and 50 nM bafilomycin A1 (Baf-A1) for 6 h. Images were recorded in the green and red channels, which were then superimposed. BF means its bright-filed image. Scale bar is 10 μm. **b,** The fluorescence ratio was calculated by dividing the intensities of the both channels (G/R). **c,** Distribution histograms of the G/R ratios for the control (dashed black line) and Baf-A-treated (red solid line) cells. Source data for this figure are provided as a Source Data file.

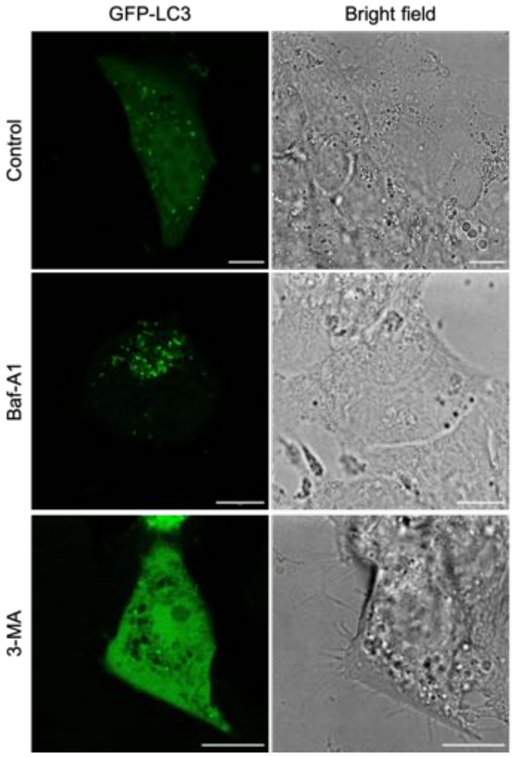

Supplementary Fig. 16. Confocal images of HepG2 cells transiently expressing GFP-LC3 cultured in the presence of each inhibitor in HBSS+ for 6 h. λ_{ex} = 473 nm, λ_{em} = 490–540 nm. Scale bar is 10 μm.

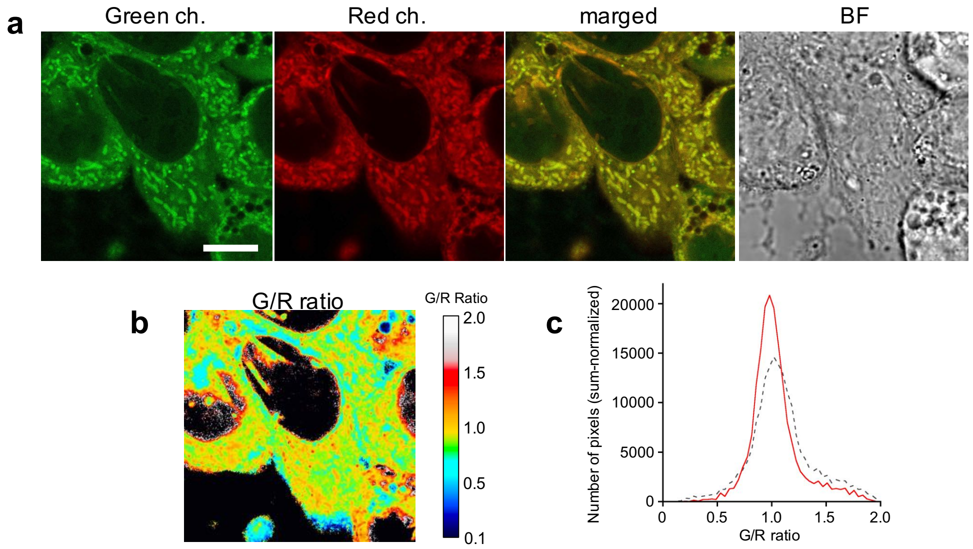

Supplementary Fig. 17. a, Confocal images of HepG2 cells incubated in HBSS+ containing 5 µM AP-C12 and 10 mM 3-methyladenine (3-MA) for 6 h. Images were recorded in the green and red channels, which were then superimposed. BF means its bright-filed image. Scale bar is 10 µm. **b,** The fluorescence ratio was calculated by dividing the intensities of the both channels (G/R). **c,** Distribution histograms of the G/R ratios for the control (dashed black line) and 3-MA-treated (red solid line) cells. Source data for this figure are provided as a Source Data file.

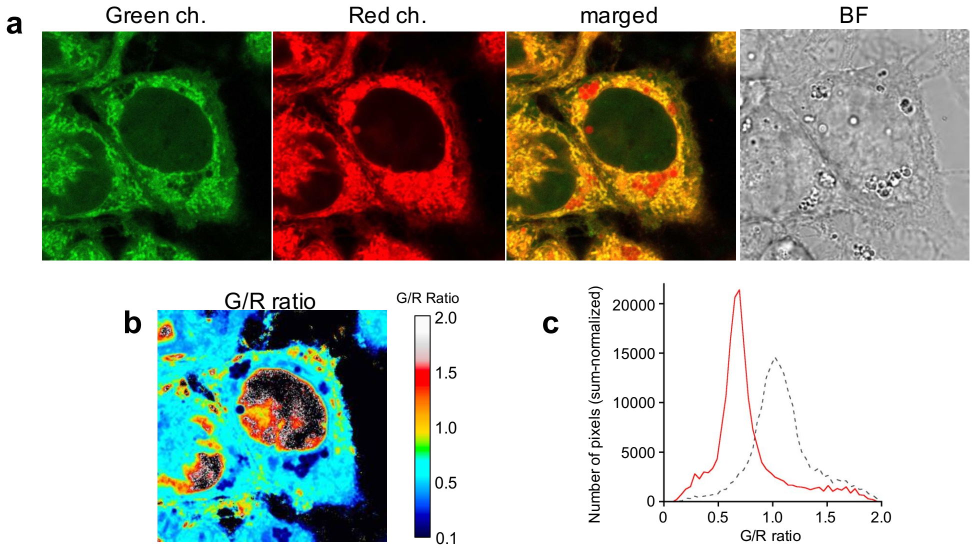

Supplementary Fig. 18. a, Confocal images of HepG2 cells incubated in HBSS+ containing 5 µM AP-C12 and 100 µM diethylumbelliferyl phosphate (DEUP) for 6 h. Images were recorded in the green and red channels, which were then superimposed. BF means its bright-filed image. Scale bar is 10 µm. **b,** The fluorescence ratio was calculated by dividing the intensities of the both channels (G/R). **c,** Distribution histograms of the G/R ratios for the control (dashed black line) and DEUP-treated (red solid line) cells. Source data for this figure are provided as a Source Data file.

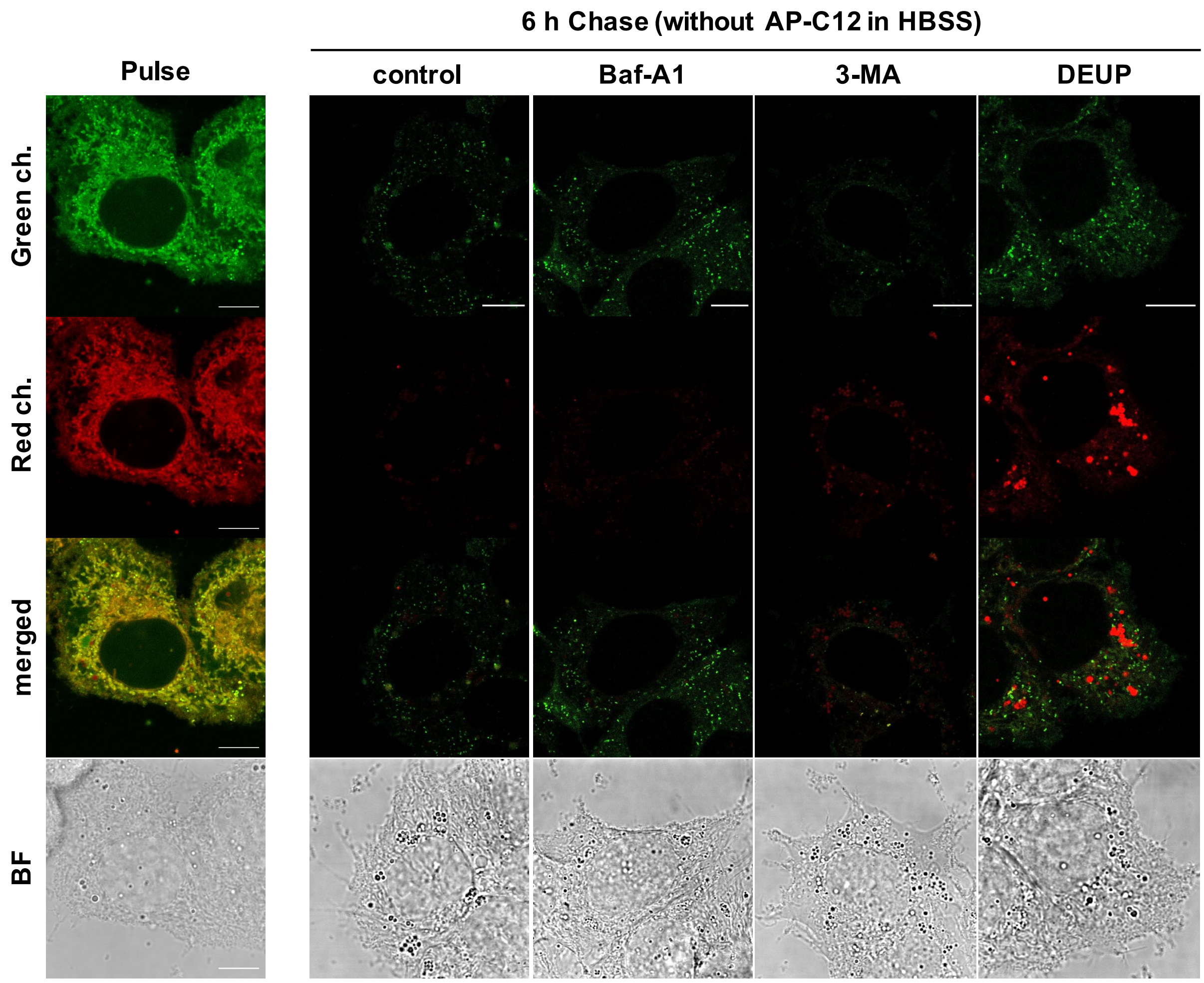

Supplementary Fig. 19. Pulse-chase assay with AP-C12. HepG2 cells were pulsed with 5 μM of AP-C12 in DMEM supplemented with XerumFree (TNC BIO) for 1 h. After the cells were rinsed with HBSS+ three times, chased in HEPES-buffered HBSS+ containing 50 nM Bafilomycin A1 (Baf-A1), 10 mM 3-methyladenine (3-MA), or 100 μM diethylumbelliferyl phosphate (DEUP) for 6 h. Scale bar is 10 μm.

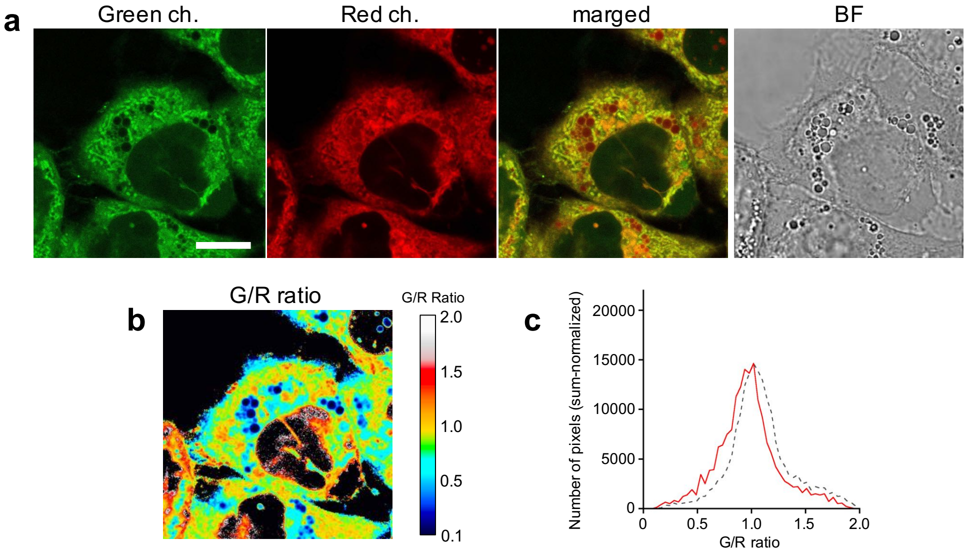

Supplementary Fig. 20. a, Confocal images of HepG2 cells incubated in HBSS+ containing 5 μM AP-C12 and 100 nM rapamycin (Rapa) for 6 h. Images were recorded in the green and red channels, which were then superimposed. BF means its bright-filed image. Scale bar is 10 μm. **b,** The fluorescence ratio was calculated by dividing the intensities of the both channels (G/R). **c,** Distribution histograms of the G/R ratios for the control (dashed black line) and Rapa-treated (red solid line) cells. Source data for this figure are provided as a Source Data file.

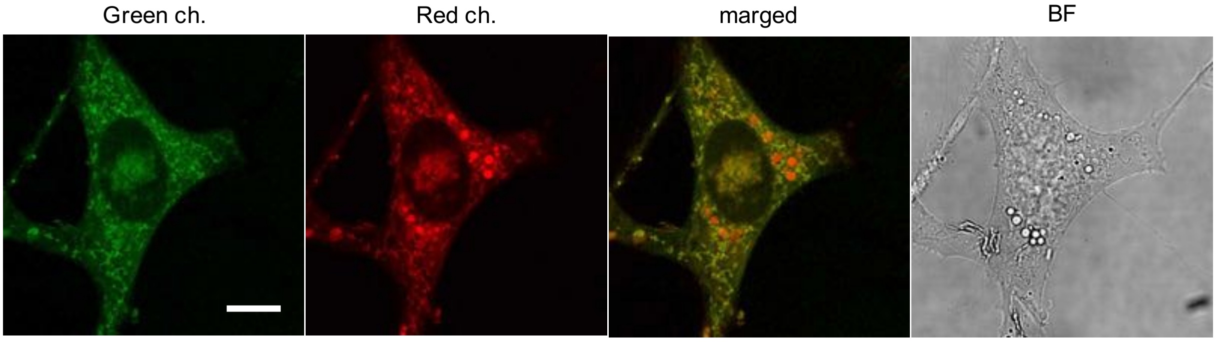

Supplementary Fig. 21. Confocal images of Atg5$^{-/-}$ mouse embryonic fibroblasts (MEFs) incubated in HBSS+ containing 5 μM AP-C12 for 6 h. Images were recorded in the green and red channels, which were then superimposed. BF means its bright-filed image. Scale bar is 10 μm.

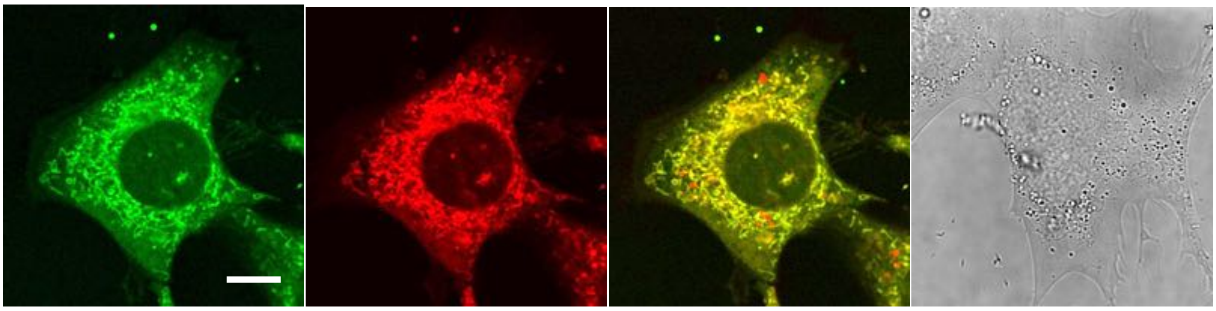

Supplementary Fig. 22. Confocal images of Atg5$^{-/-}$ MEFs incubated in HBSS+ containing 5 μM AP-C12 and 50 nM Baf-A1 for 6 h. Images were recorded in the green and red channels, which were then superimposed. BF means its bright-filed image. Scale bar is 10 μm.

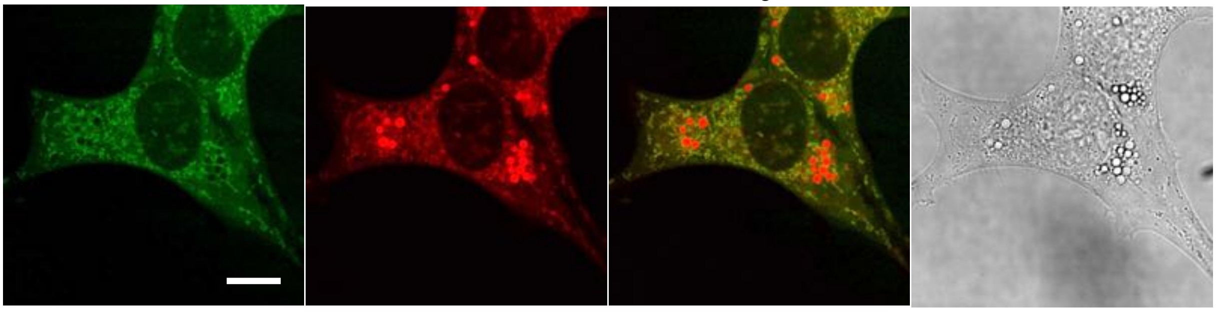

Supplementary Fig. 23. Confocal images of Atg5$^{+/+}$ MEFs incubated in HBSS+ containing 5 μM AP-C12 for 6 h. Images were recorded in the green and red channels, which were then superimposed. BF means its bright-filed image. Scale bar is 10 μm.

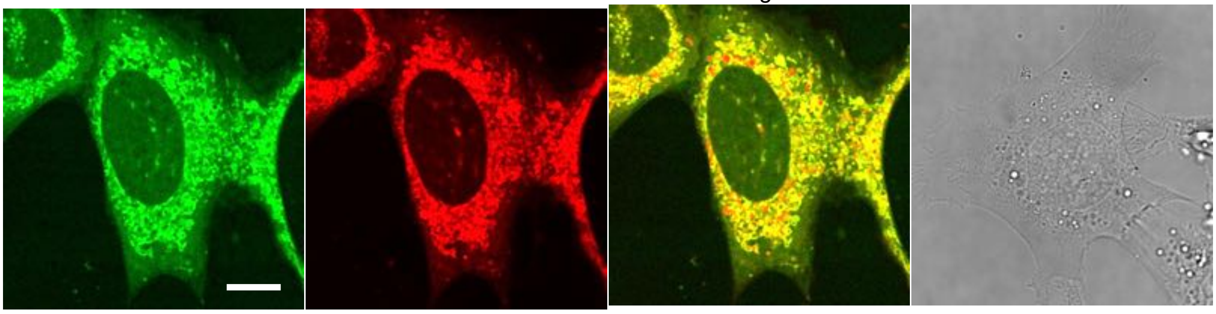

Supplementary Fig. 24. Confocal images of Atg5$^{+/+}$ MEFs incubated in HBSS+ containing 5 μM AP-C12 and 50 nM Baf-A1 for 6 h. Images were recorded in the green and red channels, which were then superimposed. BF means its bright-filed image. Scale bar is 10 μm.

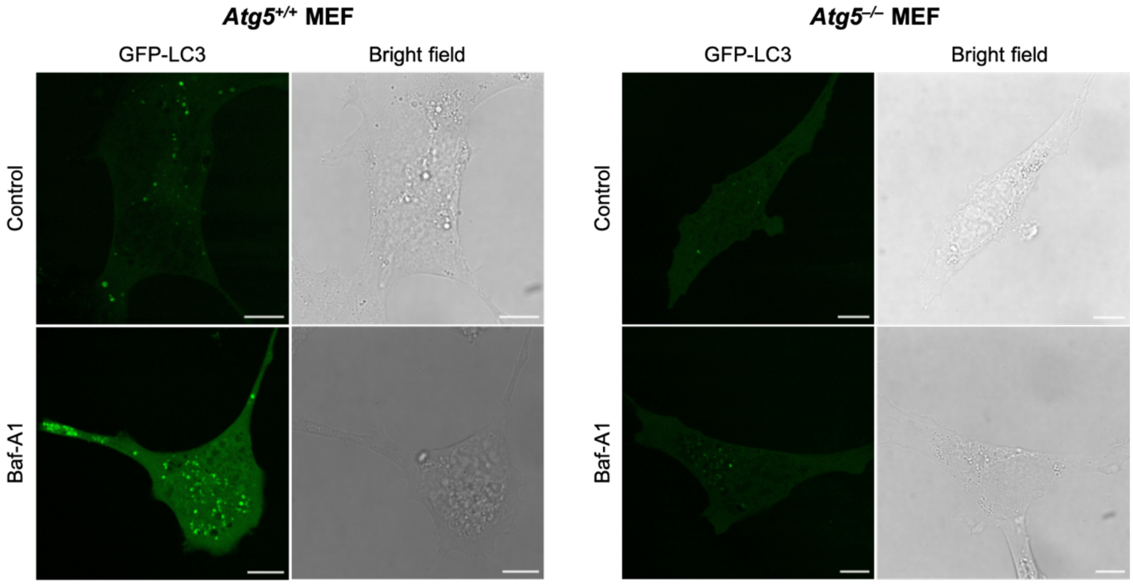

Supplementary Fig. 25. Confocal images of Atg5$^{+/+}$ (top) and Atg5$^{-/-}$ (bottom) MEFs transiently expressing GFP-LC3 cultured in HBSS+ for 6 h with/without bafilomycin A1. Scale bar is 10 μm.

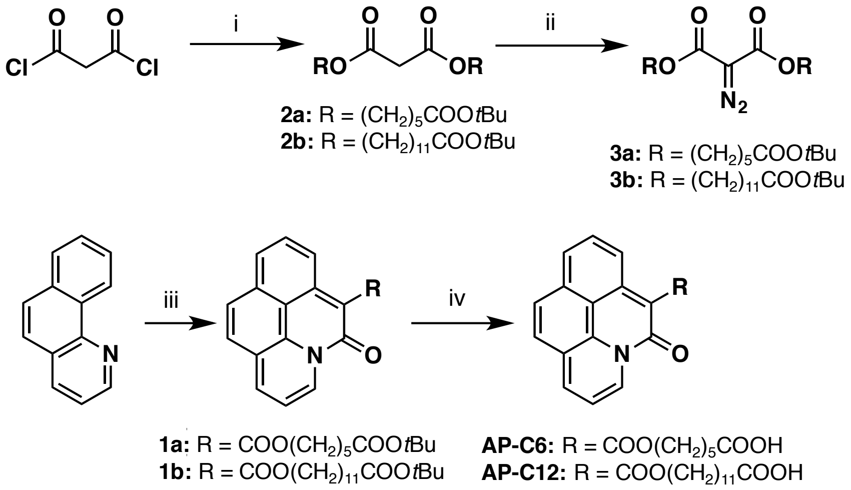

Supplementary Fig. 26. Synthetic scheme for AP-C6 and AP-C12. Reagents and conditions: (i) 6-hydroxy-t-butylhexanoate (for **2a**) or 12-hydroxy-t-butylhexanoate (for **2b**); (ii) pyridine, CH_2Cl_2, rt. then *p*-ABSA, Et_3N, $CHCl_3$, rt.; (iii) [Cp*Co(CO)I_2], $AgSbF_6$, KOAc, 2,2,2-TFE, 80–120 °C; (iv) TFA, CH_2Cl_2, rt.

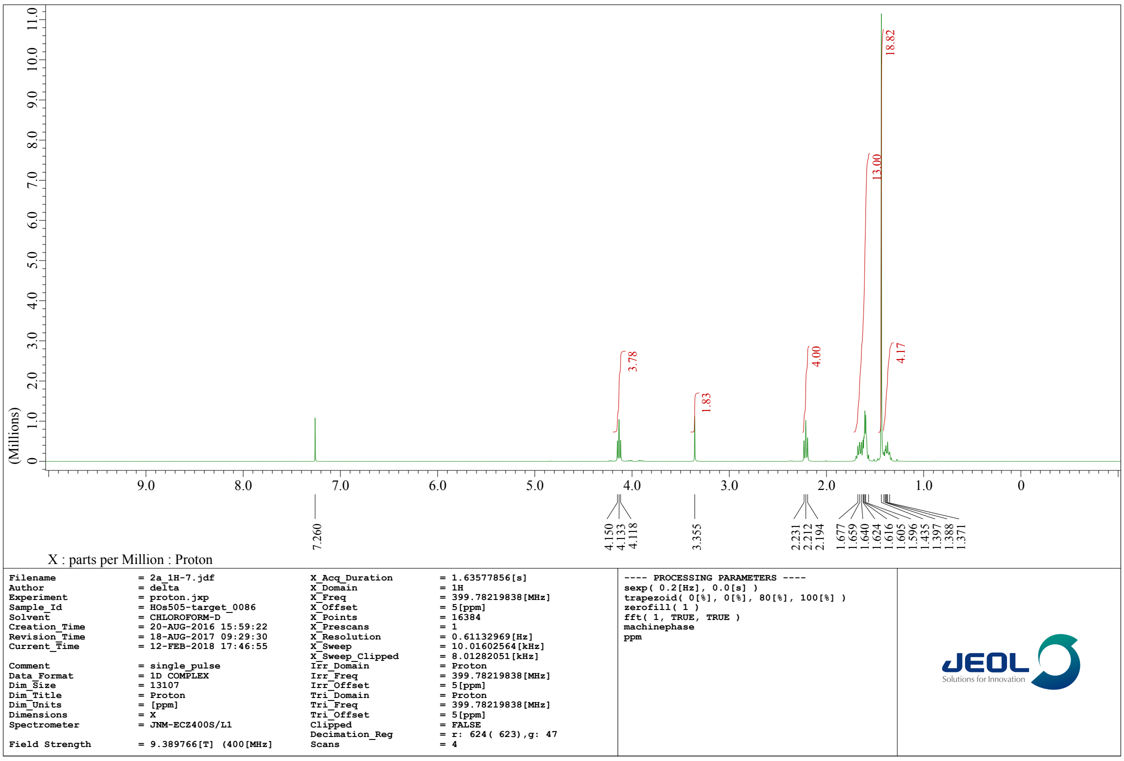

Supplementary Fig. 27 ^{1}H NMR spectrum of compound **2a** (400 MHz, $CDCl_3$).

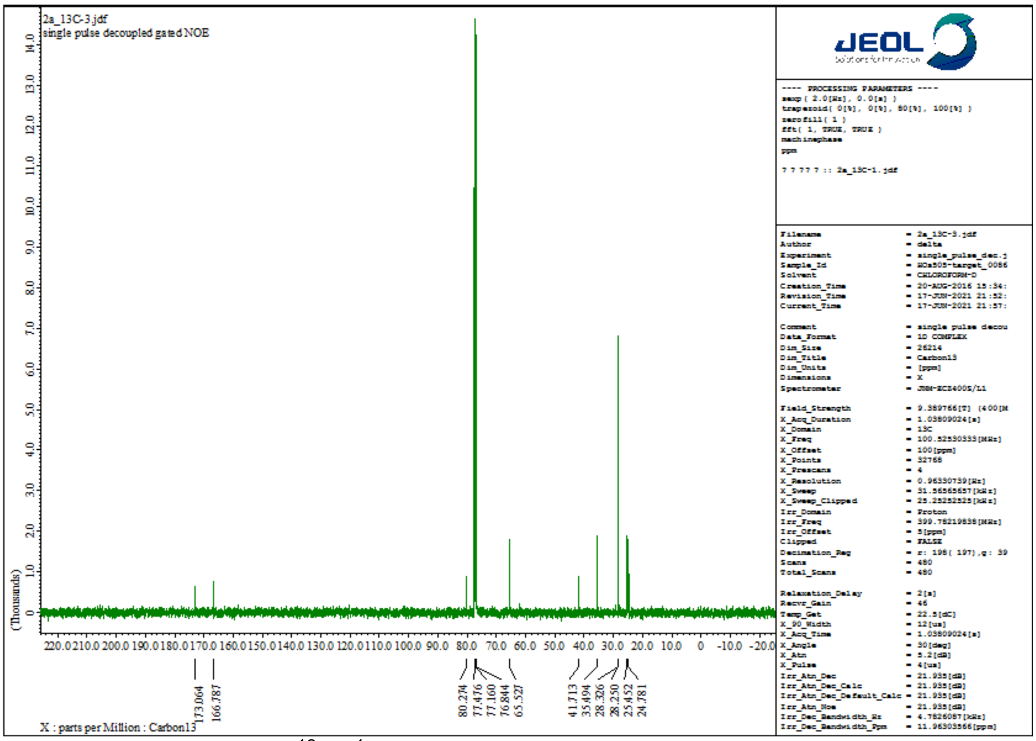

Supplementary Fig. 28 ^{13}C {^{1}H} NMR spectrum for compound **2a** (400 MHz, $CDCl_3$).

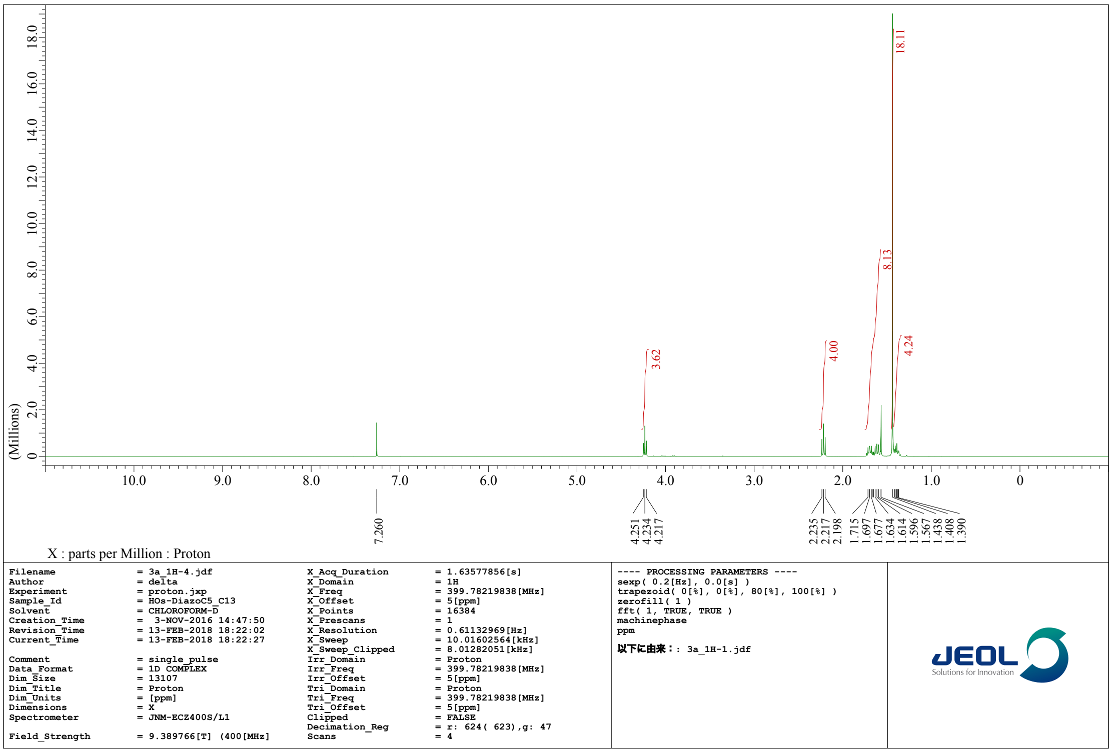

Supplementary Fig. 29 ^{1}H NMR spectrum of compound **3a** (400 MHz, $CDCl_3$).

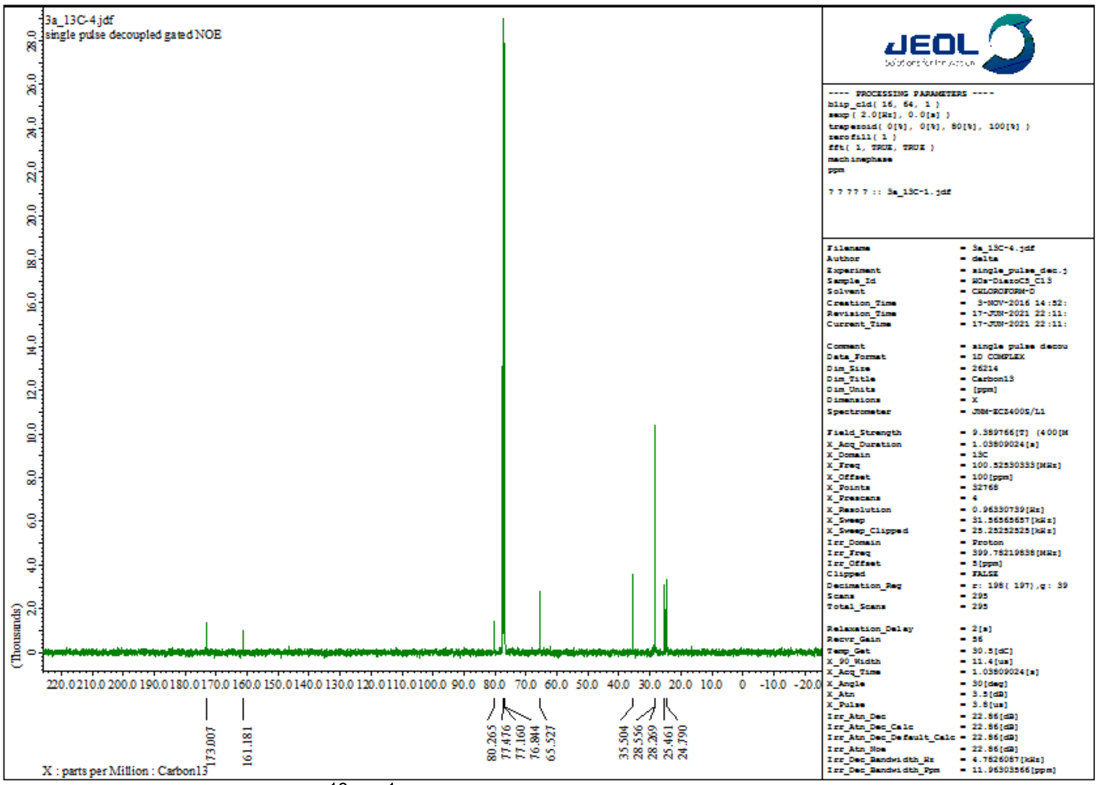

Supplementary Fig. 30 ^{13}C {^{1}H} NMR spectrum for compound **3a** (400 MHz, $CDCl_3$).

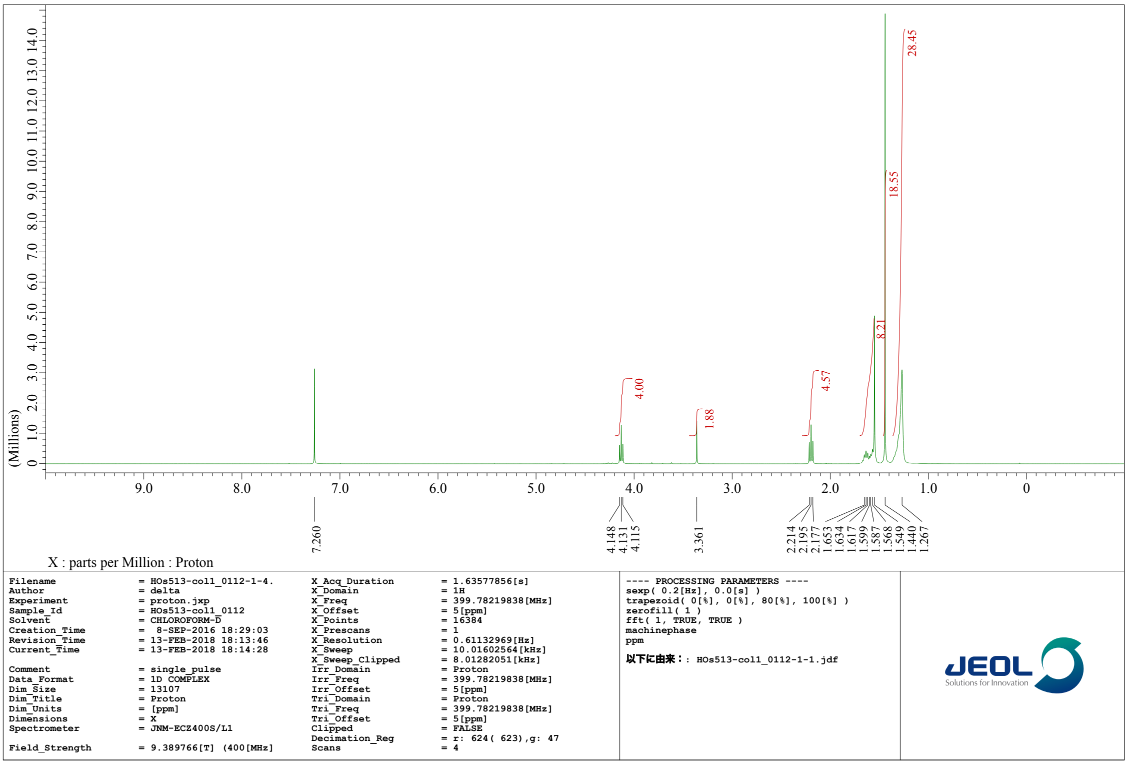

Supplementary Fig. 31 ^{1}H NMR spectrum of compound **2b** (400 MHz, $CDCl_3$).

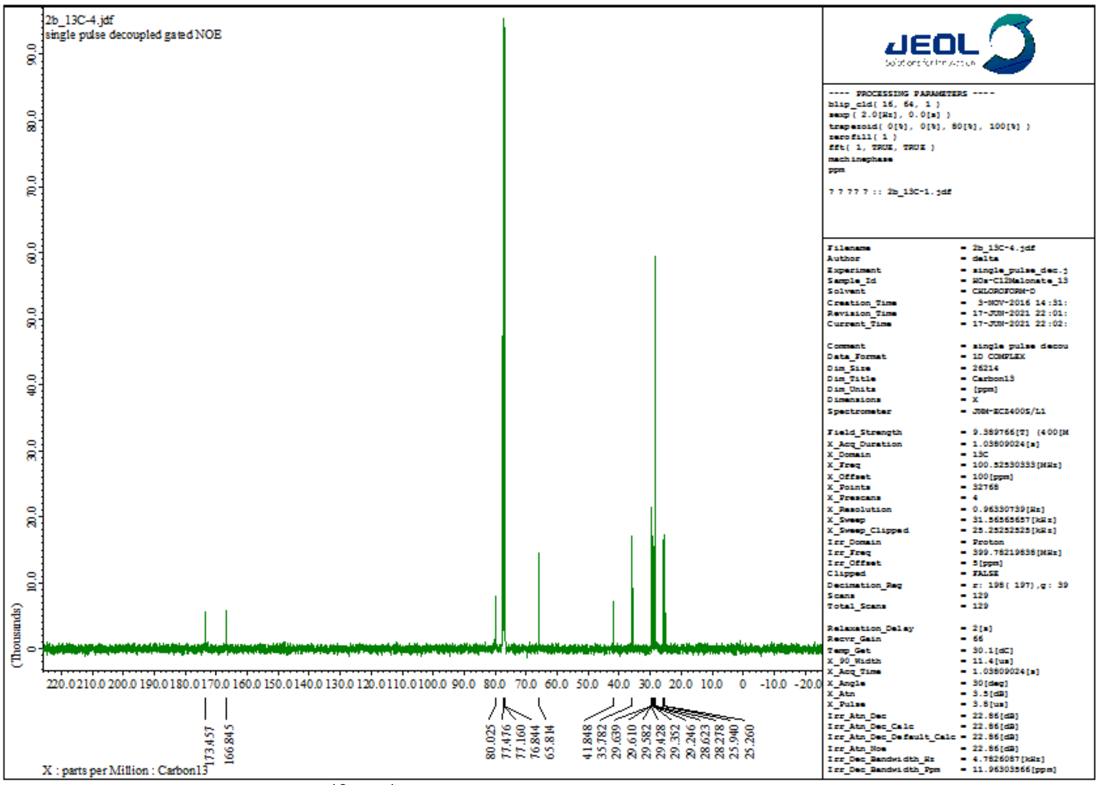

Supplementary Fig. 32 ^{13}C {^{1}H} NMR spectrum for compound **2b** (400 MHz, $CDCl_3$).

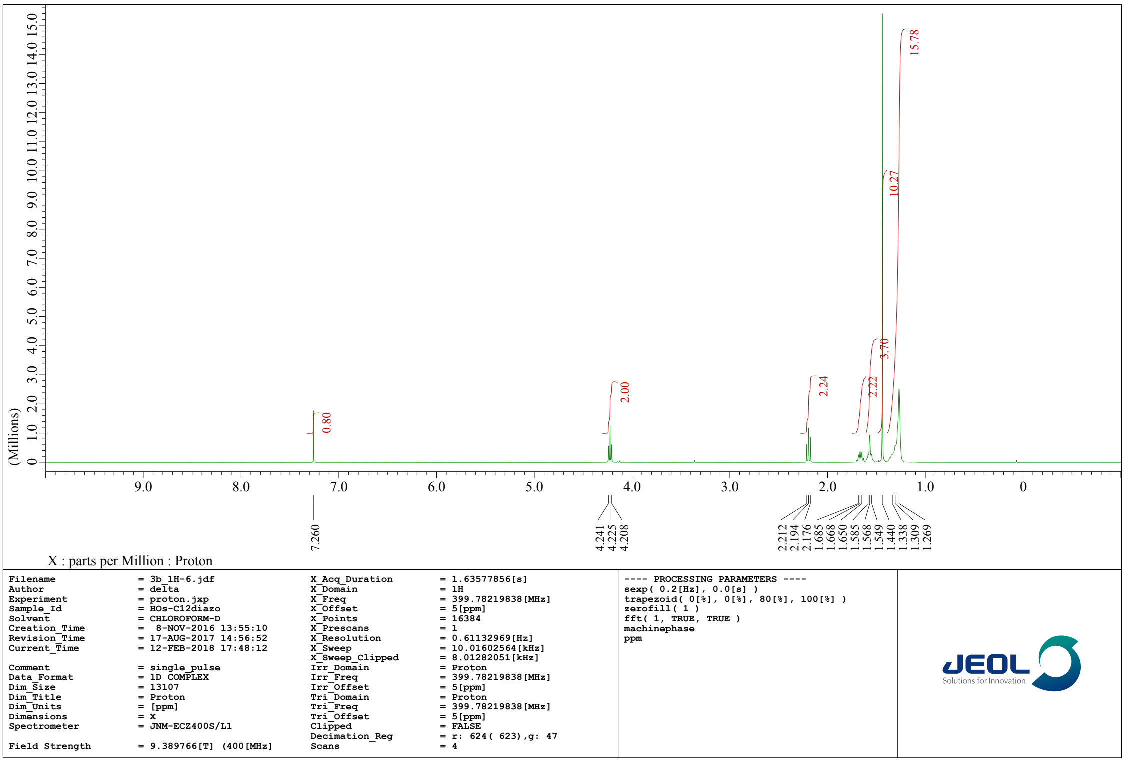

Supplementary Fig. 33 ^{1}H NMR spectrum of compound **3b** (400 MHz, $CDCl_3$).

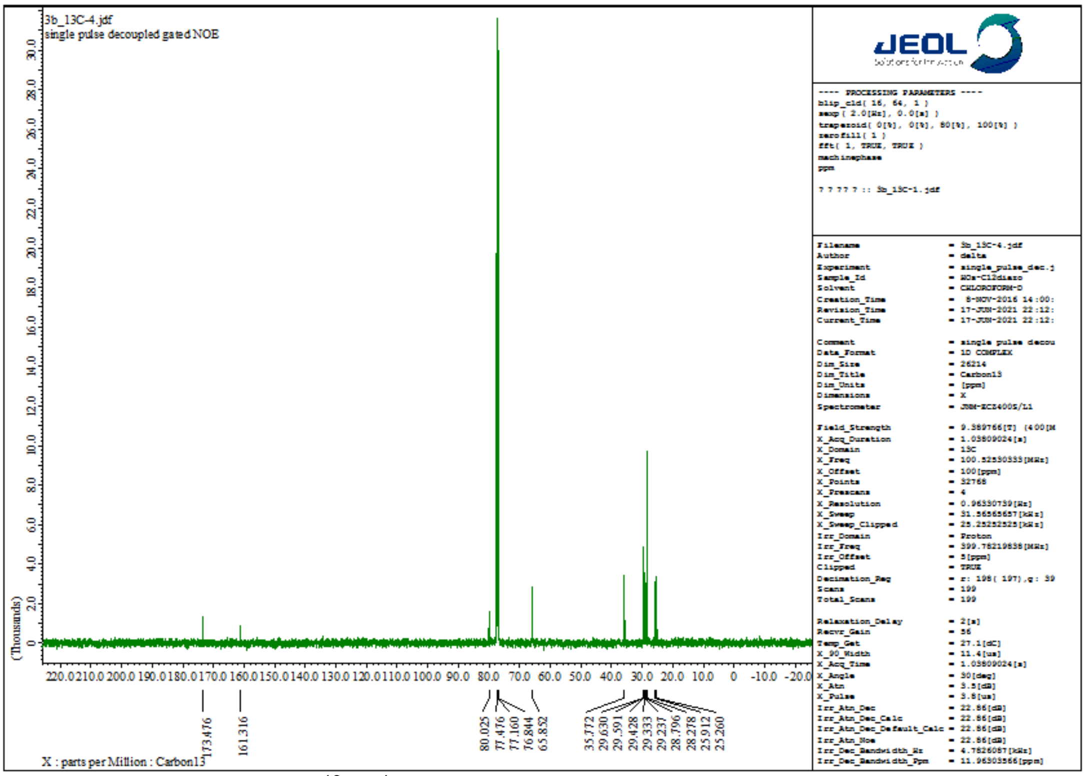

Supplementary Fig. 34 ^{13}C {^{1}H} NMR spectrum for compound **3b** (400 MHz, $CDCl_3$).

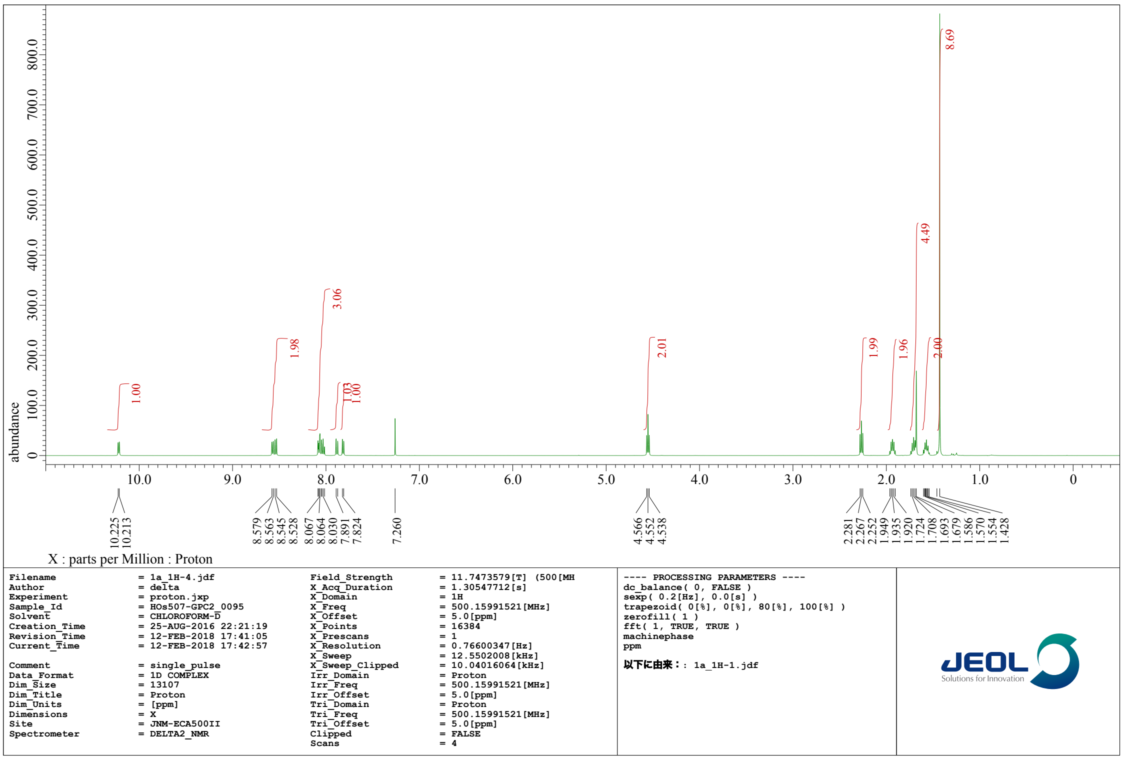

Supplementary Fig. 35 ^{1}H NMR spectrum of compound **1a** (400 MHz, $CDCl_3$).

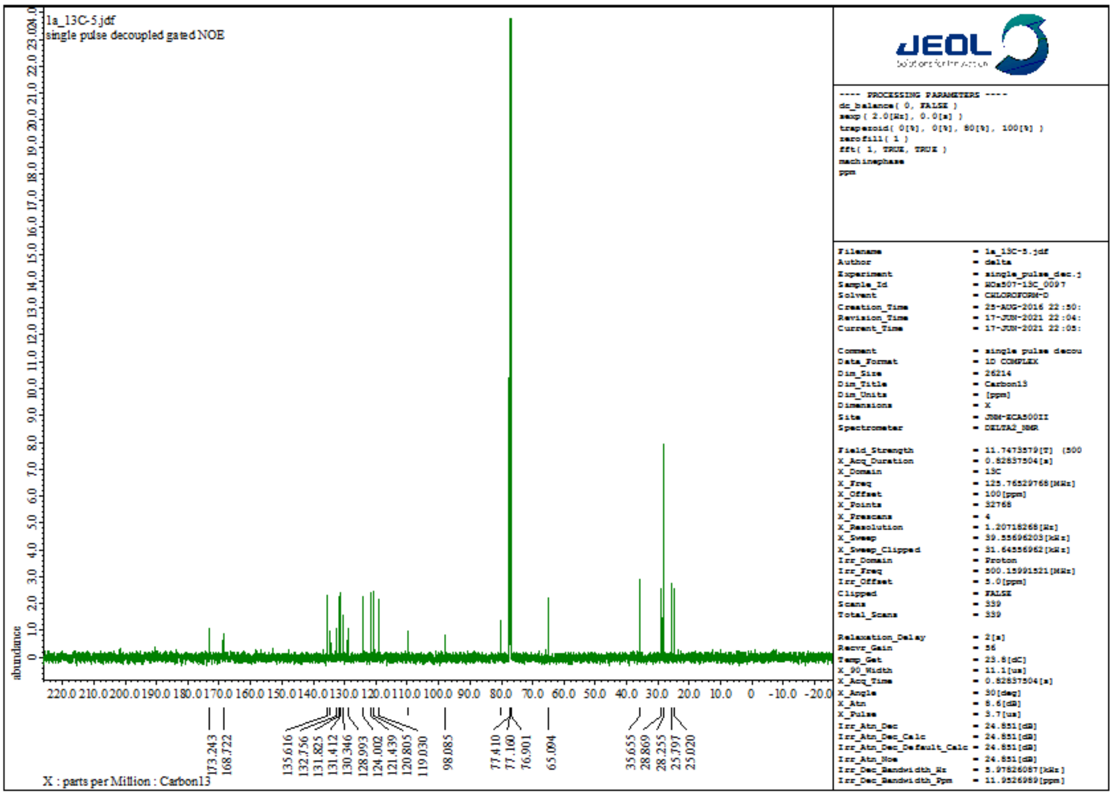

Supplementary Fig. 36 ^{13}C {^{1}H} NMR spectrum for compound **1a** (400 MHz, $CDCl_3$).

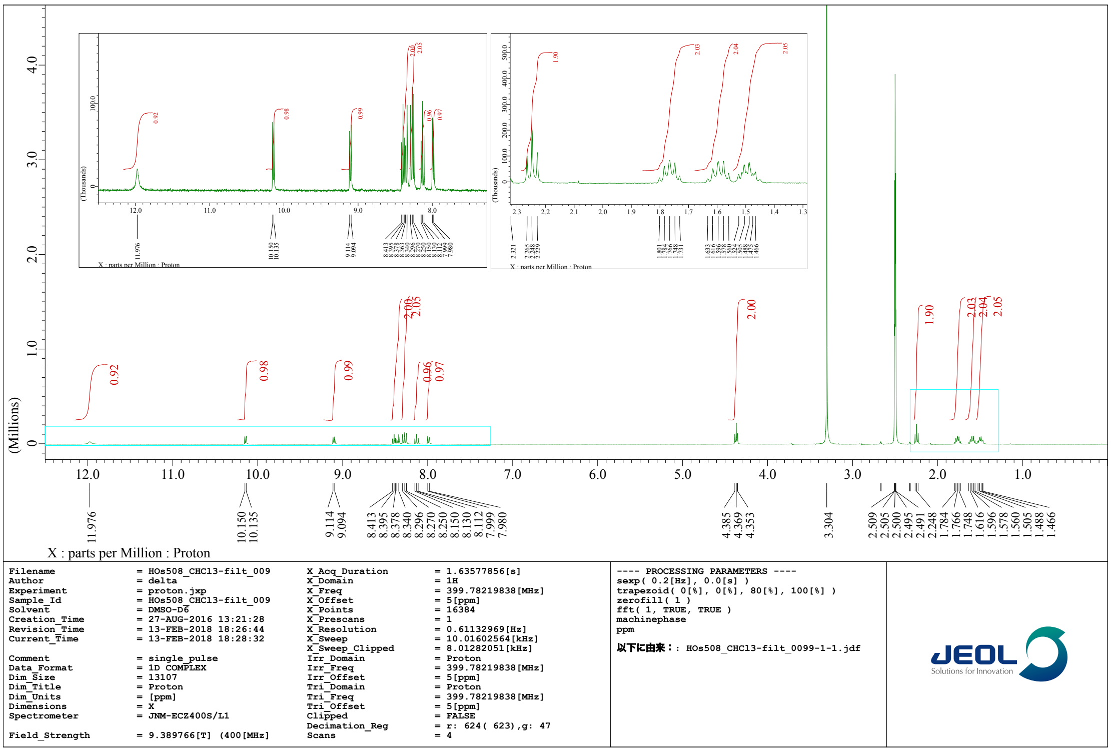

Supplementary Fig. 37 ^{1}H NMR spectrum of compound **AP-C6** (400 MHz, DMSO-d_6).

]

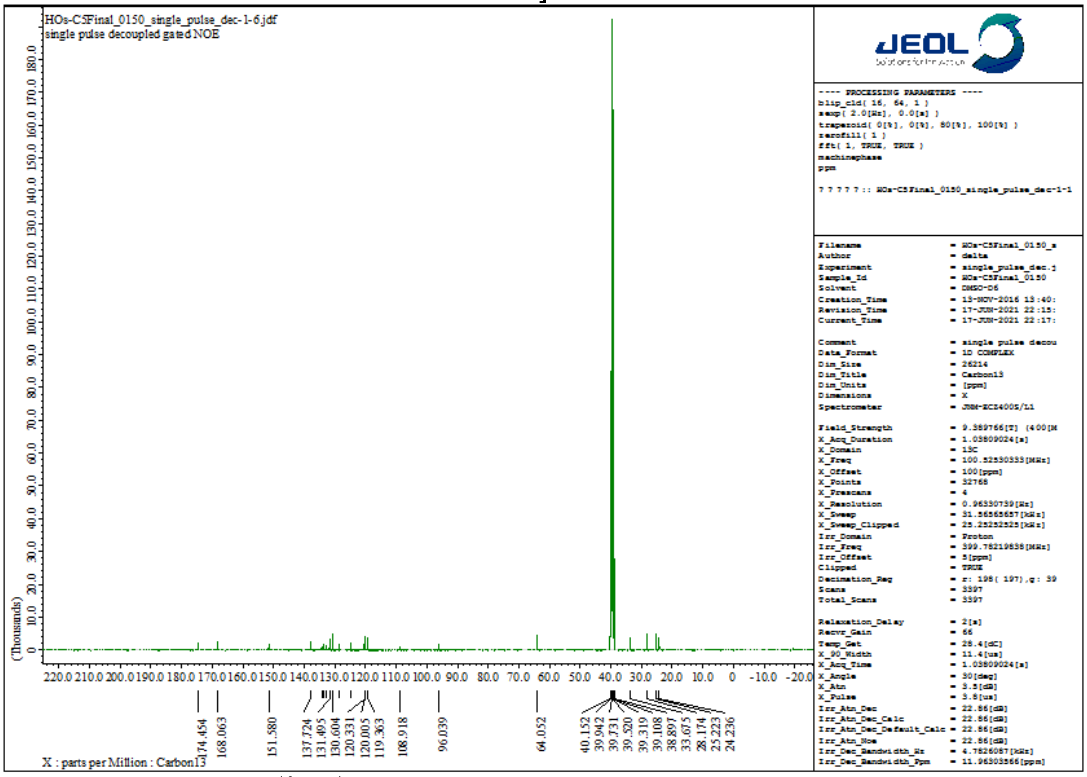

Supplementary Fig. 38 ^{13}C {^{1}H} NMR spectrum for compound **AP-C6** (400 MHz, DMSO-d_6).

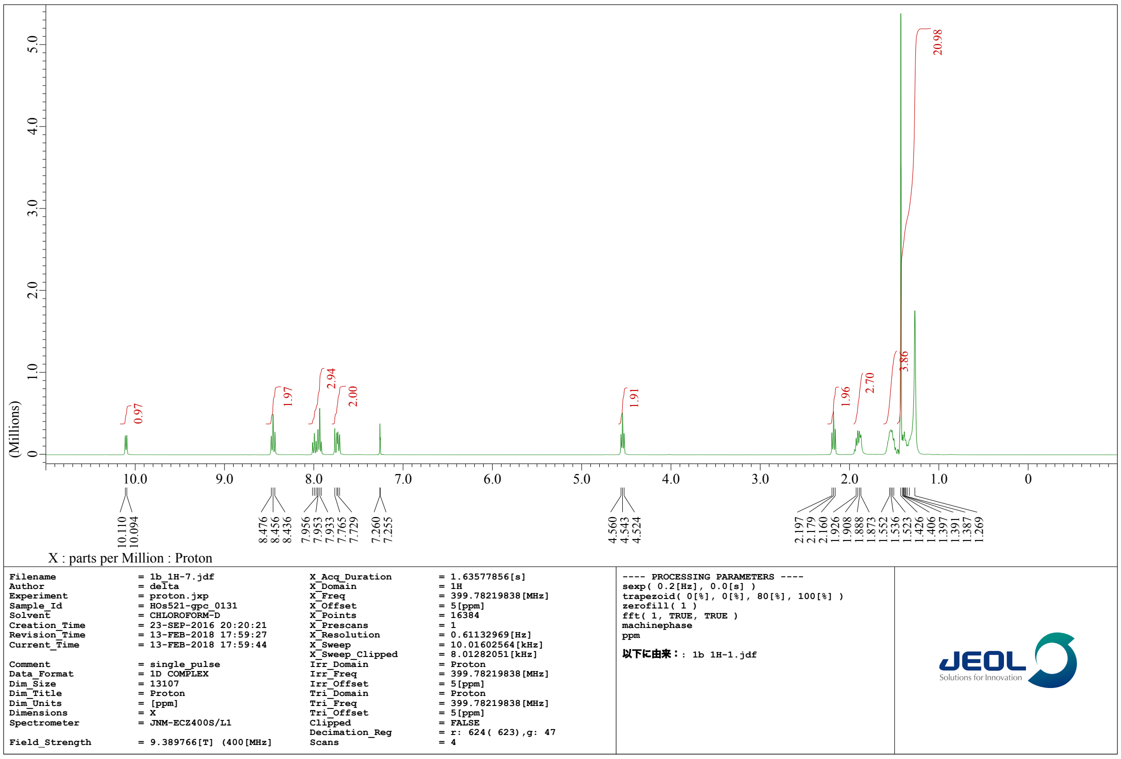

Supplementary Fig. 39 ^{1}H NMR spectrum of compound **1b** (400 MHz, $CDCl_3$).

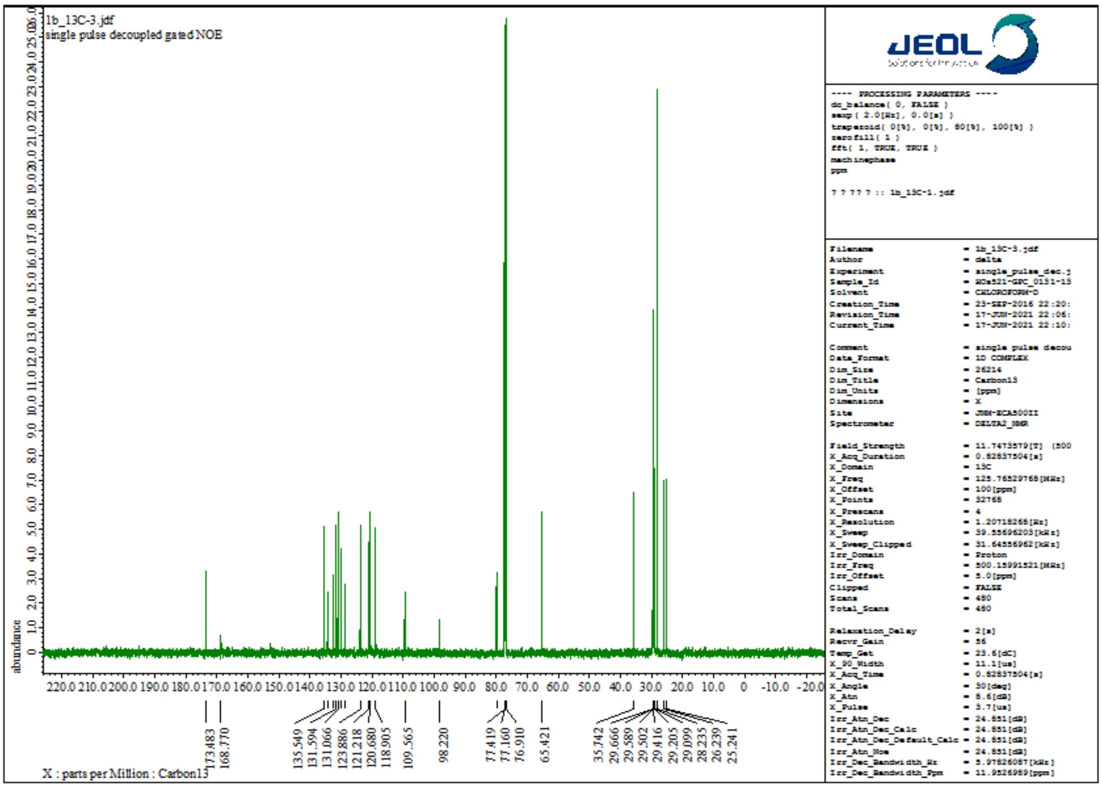

Supplementary Fig. 40 ^{13}C {^{1}H} NMR spectrum for compound **1b** (500 MHz, $CDCl_3$).

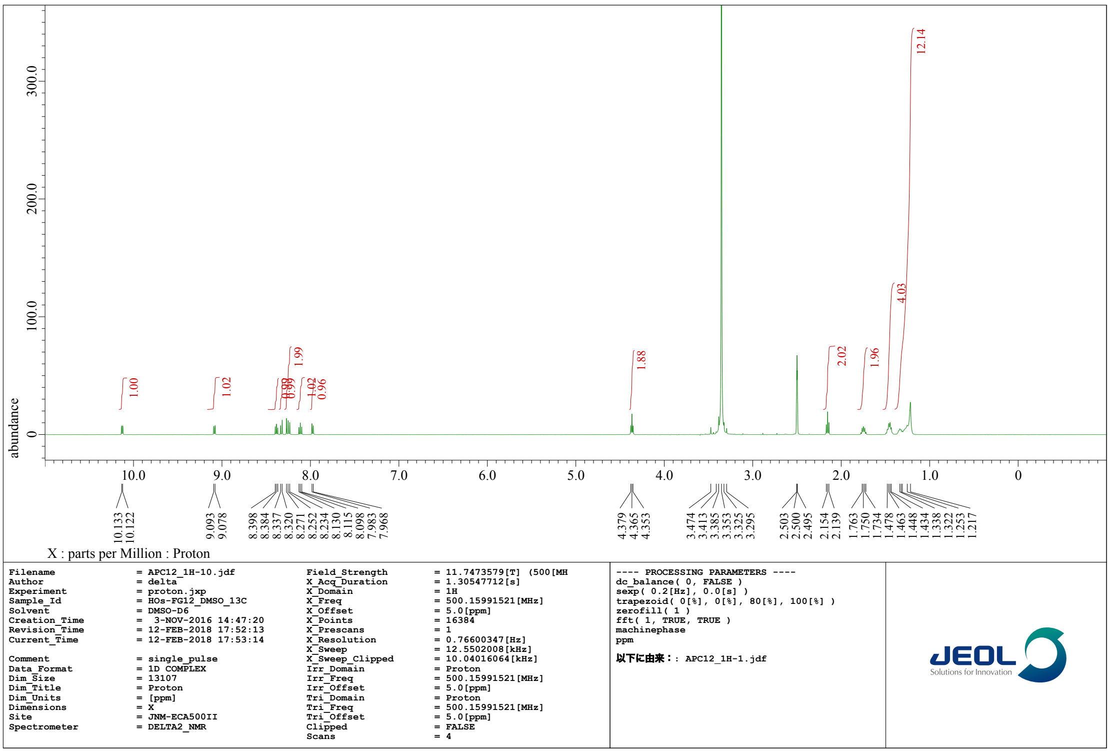

Supplementary Fig. 41 ^{1}H NMR spectrum of compound **AP-C12** (400 MHz, DMSO-d_6).

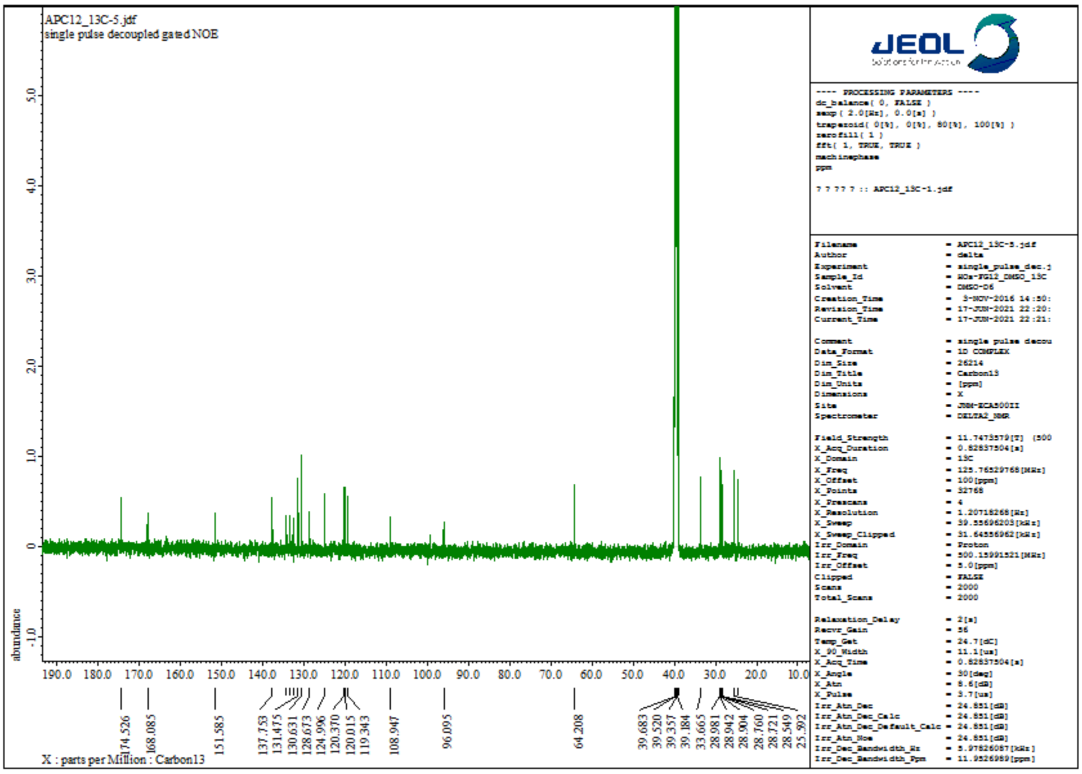

Supplementary Fig. 42 ^{13}C {^{1}H} NMR spectrum for compound **AP-C12** (500 MHz, DMSO-d_6).

Supplementary Table 1. Photophysical Data for AP-Me and dye **2–6**.

Compound	Solvent	λ_{abs} / nm[a]	ε / 10^4 M^{-1} cm^{-1}	λ_{em} / nm	Φ_F[b]
**AP-Me**	toluene	552	1.01	595	0.53
	$CHCl_3$	530	1.08	584	0.63
	CH_2Cl_2	530	1.07	585	0.56
	CH_3CN	521	1.03	583	0.63
	DMSO	523	0.99	587	0.61
	CH_3OH	496	1.04	582	0.55
	H_2O[c]	474	1.03	554	0.56
**2**	toluene	504	1.03	562	0.25
	CH_3CN	478	1.13	549	0.22
	CH_3OH	458	1.05	554	0.16
	H_2O[c]	439	0.57	535	0.16
**3**	toluene	577	0.53	687	0.04
	CH_3CN	543	0.51	677	0.04
	CH_3OH	523	0.52	665	0.03
	H_2O[c]	497	—[d]	675	0.02
**4**	toluene	460	0.83	551	0.44
	CH_3CN	445	1.21	530	0.45
	CH_3OH	439	1.23	528	0.49
	H_2O[c]	428	0.74	519	0.47
**5**	toluene	503	0.82	650	0.01
	CH_3CN	479	0.86	651	0.009
	CH_3OH	464	0.85	654	0.005
	H_2O[c]	447	0.65	650	0.003
**6**	toluene	508	0.82	572	0.26
	CH_3CN	479	1.04	562	0.20
	CH_3OH	467	1.00	570	0.16
	H_2O[c]	445	—[d]	555	0.10

[a] The longest wavelength absorption maximum. [b] Absolute fluorescence quantum yield determined by a calibrated integrating sphere system. [c] Phosphate buffer (pH 7.4) containing 1% DMSO as a co-solvent. [d] Incomplete dissolution.

Supplementary Note 1. Synthesis

General information

^{1}H and $^{13}C\{^{1}H\}$ spectra were recorded with a JEOL AL-400 spectrometer (400 MHz) for ^{1}H and 100 MHz for ^{13}C) or JEOL ECA 500 II spectrometer (125 MHz for ^{13}C) in $CDCl_3$ or DMSO-d_6. The chemical shifts in ^{1}H NMR spectra are reported in δ ppm using the residual protons of the solvents as an internal standard ($CHCl_3$ δ 7.26 and DMSO δ 2.50), and those in ^{13}C NMR spectra are reported using the solvent signals as an internal standard ($CDCl_3$ δ 77.16 and DMSO-d_6 δ 39.52). Mass spectra were measured with a Thermo Fisher Scientific Exactive spectrometer with the ESI ionization method. Thin layer chromatography was performed on plates coated with 0.25 mm thick silica gel $60F_{254}$ (Merck) and visualized by UV (254 nm) or basic permanganate stain. Column chromatography was performed using PSQ100B (Fuji Silysia Chemical). Recycling preparative gel permeation chromatography (GPC) was performed using LC-918 (Japan Analytical Industry) equipped with a polystyrene gel column (JAIGEL 1H and 2H, Japan Analytical Industry) using $CHCl_3$ as eluent. Anhydrous CH_2Cl_2 was purchased from Kanto Chemicals and further purified by Glass Contour Solvent Systems. AP-Me[1], dyes **2**–**6**[1], 6-hydroxy-*t*-butylhexanoate[2], 11-hydroxy-*t*-butyldodecanoate[3], and $[Cp^*Co(CO)I_2]$[4] were prepared according to the literature. $AgSbF_6$ and KOAc was purchased from commercial suppliers and stored in g glove box. Dry pyridine was purchased from Wako chemicals and stored over 4 Å Linde molecular sieves. All other reagents were purchased from commercial suppliers and used as received. All reactions were carried out under an argon or nitrogen atmosphere unless noted otherwise.

Bis(6-*tert*-butoxy-6-oxohexyl) malonate (**2a**)

Malonyl chloride (730 μL, 1.06 g, 7.51 mmol) was added to a solution of 6-hydroxy-*t*-butylhexanoate (3.10 g, 16.5 mmol) and anhydrous pyridine (1.6 mL, 19.8 mmol) in anhydrous CH_2Cl_2 (50 mL). The mixture was stirred for 19 h at ambient temperature and quenched with water. The aqueous layer was extracted with CH_2Cl_2, and the combined organic extract was washed with a saturated NH_4Cl solution, water, and brine. The organic layer was dried over Na_2SO_4, filtered, and concentrated under reduced pressure. Purification by silica gel column chromatography (hexane/ethyl acetate = 10:1, R_f = 0.23) afforded **2a** as a colorless oil (1.63 g, 3.68 mmol, 49%). ^{1}H NMR (400 MHz, $CDCl_3$): δ 4.13 (t, J = 6.8 Hz, 4H), 3.36 (s, 2H), 2.21 (t, J = 7.6 Hz, 4H), 1.68–1.57 (m, 8H), 1.44 (s, 18H), 1.41–1.35 (m, 4H); ^{13}C NMR (100 MHz, $CDCl_3$): δ 173.06, 166.79, 80.27, 65.53, 41.71, 35.49, 28.33, 28.25, 25.45, 24.78; HRMS (ESI, positive) calculated for $C_{23}H_{40}O_8Na$ ($[M+Na]^+$): 467.2615. Found: 467.2614.

Bis(6-*tert*-butoxy-6-oxohexyl)-2-diazomalonate (**3a**)

Triethylamine (630 μL, 4.52 mmol) was added to a suspension of **2a** (1.41 g, 3.18 mmol) and *p*-ABSA (965 mg, 4.02 mmol) in acetonitrile (15 mL). The mixture was stirred at ambient temperature for 3 days. CH_2Cl_2 was added to the suspension and filtered through a plug of celite. The filtrate was concentrated under reduced pressure, dissolved in CH_2Cl_2, and washed with a saturated aqueous NH_4Cl solution, water, and brine. The combined organic layer was dried over Na_2SO_4, filtered, and concentrated under reduced pressure. Purification by silica gel column chromatography (hexane/ethyl acetate = 5:1, R_f = 0.20) followed by GPC ($CHCl_3$) afforded **3a** as a yellow oil (1.40 g, 2.97 mmol, 93%). This reaction was performed in an ambient atmosphere: ^{1}H NMR (400 MHz, $CDCl_3$): δ 4.23 (t, J = 6.8 Hz, 4H), 2.22 (t, J = 7.6 Hz, 4H), 1.71–1.57 (m, 8H), 1.44 (s, 18H), 1.41–1.35 (m, 4H); ^{13}C NMR (100 MHz, $CDCl_3$): δ 173.01, 161.18, 80.27, 65.53, 35.50, 28.56, 28.27, 25.46, 24.79. Diazo carbon was not detected; HRMS (ESI, positive) calculated for $C_{23}H_{38}N_2O_8Na$ ($[M + Na]^+$): 493.2520; found: 493.2505.

Bis(12-*tert*-butoxy-12-oxododecyl) malonate (2b)

A solution of malonyl chloride (730 μL, 1.06 g, 7.51 mmol) in anhydrous CH_2Cl_2 (20.0 mL) was added to a solution of 12-hydroxy-*t*-butyldodecanoate (4.55 g, 16.7 mmol) and anhydrous pyridine (1.45 mL, 18.0 mmol) in anhydrous CH_2Cl_2 (20 mL) over 15 min at ambient temperature. The mixture was stirred for 16 h at ambient temperature and quenched with water. The aqueous layer was extracted with CH_2Cl_2, and the combined organic extract was washed with water and brine. The organic layer was dried over Na_2SO_4, filtered, and concentrated under reduced pressure. Purification by silica gel column chromatography (hexane/ethyl acetate = 10:1, R_f = 0.40) afforded **2b** as a colorless oil (3.65 g, 5.95 mmol, 79%). ^1H NMR (400 MHz, $CDCl_3$): δ 4.13 (t, J = 6.6 Hz, 4H), 3.36 (s, 2H), 2.20 (t, J = 7.4 Hz, 4H), 1.65–1.55 (m, 8H), 1.44 (s, 18H), 1.27 (m, br, 28H); ^{13}C NMR (100 MHz, $CDCl_3$): δ 173.46, 166.85, 80.03, 65.81, 41.85, 35.78, 29.64, 29.61, 29.58, 29.43, 29.35, 29.25, 28.62, 28.28, 25.94, 25.26, one alkylene peak is overlapped; HRMS (ESI, positive) calculated for $C_{35}H_{64}O_8Na$ ($[M+Na]^+$): 635.4493; found: 635.4484.

Bis(12-*tert*-butoxy-12-oxododecyl)-2-diazomalonate (3b)

Triethylamine (1.05 mL, 7.53 mmol) was added to a suspension of **2b** (3.09 g, 5.04 mmol) and *p*-ABSA (1.44 g, 5.60 mmol) in acetonitrile (15 mL). The mixture was stirred at ambient temperature for 40 h. Additional portion of triethylamine (500 μL, 3.59 mmol) was added, and the mixture was stirred for another 2 days. CH_2Cl_2 was added to the suspension and filtered through a plug of celite. The filtrate was concentrated under reduced pressure, dissolved in CH_2Cl_2, and washed with water and brine. The combined organic extracts were dried over Na_2SO_4, filtered, and concentrated under reduced pressure. Purification by silica gel column chromatography (hexane/ethyl acetate 9:1, R_f = 0.30) afforded **3b** as a yellow oil (2.64 g, 4.13 mmol, 82%) This reaction was performed in an ambient atmosphere: ^1H NMR (400 MHz, $CDCl_3$): δ 4.23(t, J = 6.8 Hz, 2H), 2.19 (t, J = 7.2 Hz, 2H), 1.69–1.65 (m, 2H), 1.59–1.55 (m, 2H), 1.44 (s, 18H), 1.27 (br, m, 28H); ^{13}C NMR (100 MHz, $CDCl_3$): δ 173.48, 161.32, 80.03, 65.85, 35.77, 29.63, 29.59, 29.43, 29.33, 29.24, 28.80, 28.28, 25.91, 25.26. Diazo carbon was not detected, and one alkylene peak is overlapped; HRMS (ESI, positive)

calculated for $C_{35}H_{62}N_2O_8Na$ ([*M* + Na]$^+$): 661.4398; found: 661.4388.

6-*tert*-Butoxyl-6-oxohexyl 3a-Azapyren-4-one-5-carboxylate (1a)

$AgSbF_6$ (20.6 mg, 60 μmol) and KOAc (11.8 mg, 120 μmol) was added into an oven-dried Schlenk flask under an argon atmosphere. Then, [Cp*Co(CO)I_2] (14.3 mg, 30 μmol), benzo[*h*]quinoline (109 mg, 0.608 mmol), and **3a** (419 mg, 0.890 mmol) in 2,2,2-trifluoroethanol (6 mL) was added. The resulting suspension was stirred at 80 °C for 16 h. The mixture was concentrated under reduced pressure and subjected to silica gel column chromatography (CH_2Cl_2/acetone = 10:1 then 1:1, R_f = 0.55 for 1:1). Further purification by preparative GPC ($CHCl_3$) afforded **1a** as a red solid (97.9 mg, 0.226 mmol, 38%). ^{1}H NMR (400 MHz, $CDCl_3$): δ 10.22 (d, *J* = 6.0 Hz, 1H), 8.57 (d, *J* = 8.0 Hz, 1H), 8.54 (d, *J* = 8.0 Hz, 1H), 8.09-8.02 (m, 3H), 7.88 (d, *J* = 7.0 Hz, 1H), 7.82 (d, *J* = 7.0 Hz, 1H), 4.55(t, J = 5.6 Hz, 2H), 2.27 (t, *J* = 7.0 Hz, 2H), 1.94 (quint, *J* = 7.0 Hz, 2H), 1.74–1.68 (m, 2H), 1.60–1.54 (m, 2H), 1.43 (s, 9H); ^{13}C NMR (100 MHz, $CDCl_3$): δ 173.24, 168.72, 135.62, 134.51, 132.76, 131.83, 131.41, 130.35, 129.00, 124.00, 121.44, 120.81, 119.03, 109.86, 98.09, 80.15, 65.09, 35.66, 28.87, 28.26, 25.80, 25.02; HRMS (ESI, positive) calculated for $C_{26}H_{27}O_5NNa$ ([M+Na]$^+$): 456.1781. Found: 456.1779.

6-(3a-Azapyrene-4-one-5-carbonyloxy)hexanoic acid (AP-C6)

A solution of **1a** (76.2 mg, 0.176 mmol) in TFA (5 mL) and CH_2Cl_2 (5 mL) was stirred at ambient temperature for 12 h. Water was added, and the organic layer was separated. Aqueous layer was extracted with CH_2Cl_2, and an aqueous saturated $NaHCO_3$ solution was added to the combined organic extracts. Aqueous layer was washed with CH_2Cl_2, and aqueous HCl was added to acidify the aqueous layer. CH_2Cl_2 was added to dissolve the precipitate, and the aqueous layer was washed with CH_2Cl_2. Combined organic extracts were dried over Na_2SO_4, filtered, and concentrated under reduced pressure. The obtained crude product was suspended in small amounts of $CHCl_3$ and filtered to give **AP-C6** as red solid (12.3 mg, 32.6 μmol). Filtrate was concentrated under reduced pressure, and suspended in small amounts of CH_2Cl_2. The suspension was filtered, and solids were collected to give another crop of **AP-C6**

as red solid (22.7 mg, 60.1 μmol; total yield 53%). Mp. 228 °C (decomp.) ^1H NMR (400 MHz, DMSO-d_6): δ 11.98 (s, 1H), 10.14 (d, J = 6.0 Hz, 1H), 9.10 (d, J = 8.0 Hz, 1H), 8.41-8.36 (m, 2H), 8.30-8.25 (m, 2H), 8.13 (t, J = 7.6 Hz, 1H), 7.99 (d, J = 7.6 Hz, 1H), 4.37 (t, J = 6.4 Hz, 2H), 2.25 (t, J = 7.2 Hz, 2H), 1.76 (quint, J = 7.2 Hz, 2H), 1.63–1.56 (m, 2H), 1.52–1.45 (m, 2H); ^{13}C NMR (100 MHz, DMSO-d_6): δ 174.45, 168.06, 151.58, 137.72, 134.28, 133.55, 132.66, 131.50, 130.60, 128.63, 124.96, 120.33, 120.01, 119.36, 108.92, 96.04, 64.05, 33.68, 28.17, 25.22, 24.24. One alkylene peak is overlapped; HRMS (ESI, positive) calculated for $C_{22}H_{19}O_5NNa$ ([M+Na]$^+$): 400.1155; found: 400.1153.

12-*tert*-Butoxyl-12-oxododecyl 3a-azapyren-4-one-5-carboxylate (1b)

$AgSbF_6$ (35.0 mg, 0.102 mmol) and KOAc (20.3 mg, 0.207 mmol) was added into an oven-dried Schlenk flask under an argon atmosphere. Then, [Cp*Co(CO)I_2] (23.8 mg, 50 μmol), benzo[*h*]quinoline (91.6 mg, 0.511 mmol), and **3b** (478 mg, 0.749 mmol) in TFE (5 mL) was added. The resulting suspension was stirred at 80 °C for 16 h. The mixture was concentrated under reduced pressure and subjected to silica gel column chromatography (CH_2Cl_2/acetone = 20:1 then 4:1, R_f = 0.38 for 4:1). Further purification by preparative GPC ($CHCl_3$) afforded **1b** as a red solid (80.1 mg, 0.155 mmol, 30%). ^1H NMR (400 MHz, $CDCl_3$): δ 10.10 (d, J = 6.4 Hz, 1H), 8.46 (t, J = 7.2 Hz, 1H), 8.01–7.93 (m, 3H), 7.77-7.71 (m, 2H), 4.54 (t, J = 7.2 Hz, 2H), 2.18 (t, J = 7.2 Hz, 2H), 1.93–1.87 (m, 2H), 1.55–1.45 (m, 2H), 1.43–1.27 (m, 21H); ^{13}C NMR (125 MHz, $CDCl_3$): δ 173.48, 168.77, 135.55, 134.46, 134.28, 132.54, 131.59, 131.07, 130.09, 128.72, 123.89, 121.22, 120.68, 118.91, 109.57, 98.22, 79.98, 65.42, 35.74, 29.67, 29.59, 29.50, 29.42, 29.21, 29.10, 28.24, 26.24, 25.24. One alkylene peak is overlapped; HRMS (ESI, positive) calculated for $C_{32}H_{39}NO_5Na$ ([M+Na]$^+$): 540.2720; found: 540.2718.

12-(3a-Azapyrene-4-one-5-carbonyloxy)dodecanoic acid (**AP-C12**)

A solution of **1b** (54.6 mg, 0.106 mmol) in TFA (2 mL) and CH_2Cl_2 (2 mL) was stirred at ambient temperature for 12 h. Water was added, and the organic layer was separated. Aqueous layer was extracted with CH_2Cl_2, and combined organic extracts

were washed with brine, dried over Na_2SO_4, filtered, and concentrated under reduced pressure. The obtained crude product was suspended in small amounts of CH_2Cl_2 and filtered to give red solids. The obtained solids were dried in an oven (100 °C) to afford **AP-C12** (24.9 mg, 53.9 μmol, 51%) as a red solid. Mp. 190.2–190.8 °C. ^{1}H NMR (400 MHz, DMSO-d_6): δ 10.13 (d, J = 4.4 Hz, 1H), 9.09 (d, J = 6.0 Hz, 1H), 8.38 (t, J = 5.6 Hz, 1H), 8.33 (d, J = 6.8 Hz, 1H), 8.25 (t, J = 7.4 Hz, 2H), 8.12 (t, J = 6.4 Hz, 1H), 7.98 (d, J = 6.0 Hz, 1H), 4.37 (t, J = 5.2 Hz, 2H), 2.15 (t, J = 6.0 Hz, 2H), 1.76–1.73 (m, 2H), 1.48–1.43 (m, 4H), 1.34–1.22 (m, 12H); ^{13}C NMR (125 MHz, DMSO-d_6): δ 174.53, 168.09, 151.59, 137.75, 134.31, 133.57, 132.70, 131.48, 130.63, 128.67, 125.00, 120.37, 120.02, 119.34, 108.95, 96.10, 64.21, 33.67, 28.94, 28.90, 28.76, 28.72, 28.55, 25.59, 24.51. One alkylene peak is overlapped; HRMS (ESI, negative) calculated for $C_{28}H_{30}O_5N$ ([M–H]$^-$): 460.2129; found: 460.2138.

References

1. Zhao, D., Kim, J. H., Stegemann, L., Strassert, C. A. & Glorius, F. Cobalt(Ⅲ)-catalyzed directed C-H coupling with diazo compounds: Straight forward access towards extended π-systems. *Angew. Chem. Int. Ed*. **54**, 4508–4511 (2015).
2. Larock, R. C. & Leach, D. R. Organopalladium approaches to prostaglandins. 3. Synthesis of bicyclic and tricyclic 7-oxaprostaglandin endoperoxide analogs via oxypalladation of norbornadiene. *J. Org. Chem.* **49**, 2144–2148 (1984).
3. Noguchi, H., Aoyama, T. & Shioiri, T. Total synthesis of analogs of topostin B, A DNA topoisomerase I inhibitor. Part 1. Synthesis of fragments of topostin B-1 analogs. *Tetrahedron* **51**, 10531–10544 (1995).
4. Sun, B., Yoshino, T., Matsunaga, S. & Kanai, M. Air-stable carbonyl(pentamethylcyclopentadienyl)cobalt diiodide complex as a precursor for cationic (pentamethylcyclopentadienyl)cobalt(Ⅲ) catalysis: application for directed C-2 selective C-H amidation of indoles. *Adv. Synth. Catal*. **356**, 1491–1495 (2014).
