# Supplementary material for: A negative-solvatochromic fluorescent probe for visualizing intracellular distributions of fatty acid metabolites
Source: Nat Commun. 2022 May 9;13:2533. doi: 10.1038/s41467-022-30153-6 (PMC9085894; doi:10.1038/s41467-022-30153-6)
Supplement: Supplementary file 3 — Reporting summary [file 41467_2022_30153_MOESM3_ESM.pdf]

nature portfolio

Corresponding author(s): Masayasu Taki, Shigehiro Yamaguchi, Frank Glorius

Last updated by author(s): Apr 8, 2022

Reporting Summary

Nature Portfolio wishes to improve the reproducibility of the work that we publish. This form provides structure for consistency and transparency in reporting. For further information on Nature Portfolio policies, see our Editorial Policies and the Editorial Policy Checklist.

Please do not complete any field with "not applicable" or n/a. Refer to the help text for what text to use if an item is not relevant to your study.
For final submission: please carefully check your responses for accuracy; you will not be able to make changes later.

Statistics

For all statistical analyses, confirm that the following items are present in the figure legend, table legend, main text, or Methods section.

n/a	Confirmed	
☐	☒	The exact sample size (*n*) for each experimental group/condition, given as a discrete number and unit of measurement
☐	☒	A statement on whether measurements were taken from distinct samples or whether the same sample was measured repeatedly
☐	☒	The statistical test(s) used AND whether they are one- or two-sided<br>*Only common tests should be described solely by name; describe more complex techniques in the Methods section.*
☒	☐	A description of all covariates tested
☒	☐	A description of any assumptions or corrections, such as tests of normality and adjustment for multiple comparisons
☐	☒	A full description of the statistical parameters including central tendency (e.g. means) or other basic estimates (e.g. regression coefficient) AND variation (e.g. standard deviation) or associated estimates of uncertainty (e.g. confidence intervals)
☐	☒	For null hypothesis testing, the test statistic (e.g. *F*, *t*, *r*) with confidence intervals, effect sizes, degrees of freedom and *P* value noted<br>*Give P values as exact values whenever suitable.*
☒	☐	For Bayesian analysis, information on the choice of priors and Markov chain Monte Carlo settings
☒	☐	For hierarchical and complex designs, identification of the appropriate level for tests and full reporting of outcomes
☒	☐	Estimates of effect sizes (e.g. Cohen's *d*, Pearson's *r*), indicating how they were calculated

Our web collection on statistics for biologists contains articles on many of the points above.

Software and code

Policy information about availability of computer code

Data collection	Microscope: Leica SP8 gSTED controlled by LAS X 3.5.5.19976; Zeiss LSM 780 controlled by ZEN 2012 SP1; Olympus FV10i-DOC controlled by FV10-ASW (V 1.7a).<br>Spectrometer: FluoroMax-4 spectrometer, FluorEssence (HORIBA); Agilent UV-visible ChemStation (Agilent)<br>Quantum yield: Quantaurus-QY C11347-02 (Hamamatsu Photonics)<br>Dynamic Light Scattering: Zetasizer Nano-ZS (Malvern Panalytical)<br>Laser Scanner: Typhoon FLA9500 (GE HealthCare)
Data analysis	ImageJ (v1.53), Excel 2016 (v16.16.17), KaleidaGraph 4.5 (v4.5.3), Huygens Professional (v 18.10.0p8 64b), Python 3.7.9

For manuscripts utilizing custom algorithms or software that are central to the research but not yet described in published literature, software must be made available to editors and reviewers. We strongly encourage code deposition in a community repository (e.g. GitHub). See the Nature Portfolio guidelines for submitting code & software for further information.

Data

Policy information about availability of data

All manuscripts must include a data availability statement. This statement should provide the following information, where applicable:

- Accession codes, unique identifiers, or web links for publicly available datasets
- A description of any restrictions on data availability
- For clinical datasets or third party data, please ensure that the statement adheres to our policy

The authors declare that all data supporting the findings of this study are available within the manuscript and its Supplementary Information and are available from the corresponding author upon request.

Field-specific reporting

Please select the one below that is the best fit for your research. If you are not sure, read the appropriate sections before making your selection.

☒ Life sciences ☐ Behavioural & social sciences ☐ Ecological, evolutionary & environmental sciences

Life sciences study design

All studies must disclose on these points even when the disclosure is negative.

Sample size	All quantitative imaging experiments were performed on replicate samples or fields of view as reported in the figure legends.
Data exclusions	No data were excluded from the analyses.
Replication	All imaging data were repeated independently at least twice and showed similar results.
Randomization	This study does not require the allocation of samples to different experimental groups. Thus, randomization was not necessary and was not performed.
Blinding	This study does not involve comparisons between experimental groups. Thus, blinding was not necessary.

Reporting for specific materials, systems and methods

We require information from authors about some types of materials, experimental systems and methods used in many studies. Here, indicate whether each material, system or method listed is relevant to your study. If you are not sure if a list item applies to your research, read the appropriate section before selecting a response.

Materials & experimental systems

n/a	Involved in the study	
☒	☐	Antibodies
☐	☒	Eukaryotic cell lines
☒	☐	Palaeontology and archaeology
☒	☐	Animals and other organisms
☒	☐	Human research participants
☒	☐	Clinical data
☒	☐	Dual use research of concern

Methods

n/a	Involved in the study	
☒	☐	ChIP-seq
☒	☐	Flow cytometry
☒	☐	MRI-based neuroimaging

Eukaryotic cell lines

Policy information about cell lines

Cell line source(s)	3T3-L1 (JCRB Cell Bank), HepG2 (RIKEN BRC Cell Bank), Atg5+/+ MEF (RIKEN BRC Cell Bank), Atg5–/– MEF (RIKEN BRC Cell Bank)
Authentication	None of the cell lines used in this study was authenticated by the authors.
Mycoplasma contamination	The cell lines were not tested for Mycoplasma contamination.
Commonly misidentified lines (See ICLAC register)	No commonly misidentified cell lines were used.

This checklist template is licensed under a Creative Commons Attribution 4.0 International License, which permits use, sharing, adaptation, distribution and reproduction in any medium or format, as long as you give appropriate credit to the original author(s) and the source, provide a link to the Creative Commons license, and indicate if changes were made. The images or other third party material in this article are included in the article's Creative Commons license, unless indicated otherwise in a credit line to the material. If material is not included in the article's Creative Commons license and your intended use is not permitted by statutory regulation or exceeds the permitted use, you will need to obtain permission directly from the copyright holder. To view a copy of this license, visit http://creativecommons.org/licenses/by/4.0/
